# Integrated Analysis of Multiple Microarray Studies to Identify Core Gene-Expression Signatures Involved in Tubulointerstitial Injury in Diabetic Nephropathy

**DOI:** 10.1155/2022/9554658

**Published:** 2022-05-10

**Authors:** Huandi Zhou, Zhifen Yang, Lin Mu, Yonghong Shi

**Affiliations:** ^1^Department of Pathology, Hebei Medical University, Shijiazhuang 050017, China; ^2^Hebei Key Laboratory of Kidney Disease, Hebei Medical University, Shijiazhuang, Hebei 050017, China; ^3^Department of Radiotherapy, Second Hospital of Hebei Medical University, Shijiazhuang, Hebei 050000, China; ^4^Gynecology and Obstetrics, The Fourth Hospital of Hebei Medical University, Shijiazhuang, Hebei 050000, China

## Abstract

Diabetic nephropathy is a leading cause of end-stage renal disease in both developed and developing countries. It is lack of specific diagnosis, and the pathogenesis remains unclarified in diabetic nephropathy, following the unsatisfactory effects of existing treatments. Therefore, it is very meaningful to find biomarkers with high specificity and potential targets. Two datasets, GSE30529 and GSE47184 from GEO based on diabetic nephropathy tubular samples, were downloaded and merged after batch effect removal. A total of 545 different expression genes screened with log2FC > 0.5 were weighted gene coexpression correlation network analysis, and green module and blue module were identified. The results of KEGG analyses both in green module and GSEA analysis showed the same two enriched pathway, focal adhesion and viral myocarditis. Based on the intersection among WGCNA focal adhesion/Viral myocarditis, GSEA focal adhesion/viral myocarditis, and PPI network, 17 core genes, ACTN1, CAV1, PRKCB, PDGFRA, COL1A2, COL6A3, RHOA, VWF, FN1, HLA-F, HLA-DPB1, ITGB2, HLA-DRA, HLA-DMA, HLA-DPA1, HLA-B, and HLA-DMB, were identified as potential biomarkers in diabetic tubulointerstitial injury and were further validated externally for expression at GSE99325 and GSE104954 and clinical feature at nephroseq V5 online platform. CMap analysis suggested that two compounds, LY-294002 and bufexamac, may be new insights for therapeutics of diabetic tubulointerstitial injury. Conclusively, it was raised that a series of core genes may be as potential biomarkers for diagnosis and two prospective compounds.

## 1. Introduction

Diabetic nephropathy (DN), affecting approximately 30-40% of patients with diabetes mellitus (DM) as a devastating microvascular complication, is the primary cause of chronic kidney disease (CKD) and end-stage renal disease (ESRD) in the world [1]. DN is characterized by progressive renal damage manifested by a deterioration in the glomerular filtration rate (GFR), progressive proteinuria, an increased serum creatinine level (SCR), a progressive urine albumin creatinine ratio (ACR), hypertension, and a high mortality rate because of complications from ESRD or cardiovascular diseases. DN patients taken in proportion of 30-47% of all patients enrolled in ESRD programs, a trend which is significantly associated with the growing incidence and mortality rates of diabetic patients [2]. Due to the high morbidity ratio and significant public health problems associated with ESRD, early diagnosis, reasonable therapeutics, and the postponement of DN onset have promising clinical implications.

In the past, DN was considered to be a glomerular disease mainly characterized by vascular damage. However, some studies have reported recently that patients with advanced DN have either substantial glomerular pathological change or proteinuria. It appears that a decline in renal function precedes traditional indicators of renal disease, such as microalbuminuria or creatinine [3, 4]. Emerging evidence supports the concept that renal tubular lesions play an important role in the occurrence and development of DN. A large number of studies have shown that the capacity for albumin reabsorption decreases after the renal tubular function is compromised, which occurs significantly earlier than the changes in renal function and glomerular filtration function. Simple renal tubular dysfunction can also cause proteinuria [3–6]. The renal tubules may act as initiators, drivers, or contributors in the early pathogenesis of DN [5]. Renal tubulopathy and renal interstitial fibrosis can be used as relatively independent factors to predict the progression of renal disease. Thus, there is an increasing need to exploring molecular alterations of renal tubules as biomarkers for develop effective early diagnosis protocols and understand the precise molecular mechanisms involved in disease progression related to therapeutic strategies in DN.

Currently, bioinformatics methods are extensively applied to the analysis of microarray data to identify differentially expressed genes (DEGs), followed by mechanism exploration. However, due to a limited sample size, reliable results may be difficult to come by during a single ChIP analysis given the high false-positive rate. In our research, four mRNA-expression profiling arrays were downloaded from the Gene Expression Omnibus (GEO), of which two datasets, GSE30529 and GSE47184, were merged after batch-effect removal to screen the DEGs and subsequent mechanisms, while other two datasets, GSE99325 and GSE104954, were regarded as validation sets, respectively. DEGs expressed in renal tubulointerstitial tissues between DN patients and normal controls were filtrated to seek prospective biomarkers. Weighted gene coexpression network analysis (WGCNA), protein-protein interaction (PPI) network, gene set enrichment analysis (GSEA), and Kyoto Encyclopedia of Genes and Genomes (KEGG)/Gene Ontology (GO) analysis were executed to identify the central biomarkers and explore the molecular mechanisms that bring about renal tubule damage. Complementally, the Nephroseq v5 online platform was used to validate the Pearson correlations between core genes and the clinical performance of DN. Possible small-molecule drugs that could reverse the major tubulointerstitial changes in DN were revealed by connectivity mapping (CMap). Conclusively, a total of 17 core genes, two crucial pathways, and 10 potential drugs (especially LY-294002 and bufexamac) were found, which can be regarded as hopeful diagnostic biomarkers and therapeutic strategies for tubulointerstitial lesions in DN, respectively.

## 2. Materials and Methods

### 2.1. Microarray Data Information and DEG Analysis

Four gene-expression datasets (GSE30529, GSE47184, GSE99325, and GSE104954) were downloaded from GEO database (http://www.ncbi.nlm.nih.gov/geo), a publicly available functional genomics database, to screen and identify candidate genes involved in tubulointerstitial injury of patients with DN. GSE30529 was performed on the GPL571 platform (Human Genome U133A 2.0 Array; Affymetrix, Santa Clara, CA, USA). GSE47184 was performed on both the GPL11670 (Human Genome U133 Plus 2.0 Array; Affymetrix) and GPL14663 (GeneChip Human Genome HG-U133A Custom CDF; Affymetrix) platforms. GSE99325 was tested on the GPL19109 (Human Genome U133 Plus 2.0 Array; Affymetrix) and GPL19184 (Human Genome U133A Array; Affymetrix) platforms. GSE104954 was tested on the GPL24120 (Human Genome U133A Array; Affymetrix) and GPL22945 (Human Genome U133 Plus 2.0 Array; Affymetrix) platforms, respectively.

Data preprocessing included probe conversion, data integration, and batch-removal effects. Genes with ≥1 probe sets or probes without corresponding gene symbols were averaged or removed, respectively. InSilicoMerging R/Bioconductor packages were used to integrate and normalize them across platforms, respectively. The DEGs in DN and normal renal tissues were screened by a cut-off criterion, adjusted *P* < 0.05 and |log2FC| > 0.5, using the linear models for microarray data (limma) R package. The heat map of DEGs was calculated and mapped using “Pheatmap” R package.

### 2.2. Construction of the Weighted Gene Coexpression Network

WGCNA is a system biology method used to describe gene-association patterns between different samples. It can be used to identify highly covarying gene sets and to identify candidate biomarker genes or therapeutic targets based on the interconnectedness of gene sets and the association between gene sets and phenotypes. A gene coexpression network was constructed for analyzing the coexpression relationship of DEGs using the WGCNA online tool, Sanger Box (http://sangerbox.com/).

WGCNA analysis can be divided into three steps, as follows: (i) select an appropriate soft threshold, (ii) determine the coexpression module, and (iii) analyze the relationship between modules and phenotypes. Briefly, genes were analyzed by both Pearson's correlation matrices and an average linkage method. Then, *β*, a soft power threshold, that may underline strong correlations between genes and penalize weak correlations, was used to convert the correlation matrix into a weighted adjacency matrix using a power function *A* *mn* = |*C* *mn*|*β* (where *C* *mn* equals = Pearson′s correlation between gene *m* and gene *n*, and *A* *mn* equals the adjacency between gene *m* and gene *n*). The adjacency was then switched into a topological overlap matrix (TOM), which was used to measure the network connectivity of a gene. The resulting TOM was based on genetic similarity of biological significance and was used to measure the coexpression relationships between genes. It was defined as the sum of its adjacency with all other genes for network gene ratio. This was followed by the calculation of corresponding dissimilarities (1-TOM). Module identification was achieved using the method of dynamic tree cutting based on hierarchical clustering with a minimum size (gene group) of 20 for the genes dendrogram. The sensitivity was set to 2. To further analyze the module, we calculated the dissimilarity of module eigen genes, chose a cut line for the module dendrogram, and merged some modules with a distance < 0.25. It should be noted that grey module is considered a gene set that could not be assigned to any module.

### 2.3. GSEA

GSEA was conducted to explore the underlying biological pathways. The 46 samples in GSE30529 + GSE47184 belonged to two groups of 28 DN samples and 18 controls and underwent enrichment analysis using the GSEA software (GSEA_4.1.0, http://software.broadhttp://institute.org/gsea/), on the JAVA version 8.0 platform. The annotated gene set c2.cp.kegg.v7.4.symbols.gmt obtained from the GSEA official website (http://www.gsea-msigdb.org/gsea/index.jsp) was chosen as the reference set to calculate enrichment score (ES). The number of permutations was set to 1000. The gene size was set to 5-500. A normalized *P* < 0.05 and a false − discovery rate (FDR) < 0.25 were considered to be statistically significant.

### 2.4. KEGG Pathway and GO Analysis

For gene set functional enrichment analysis, we used the latest KEGG pathway gene annotation obtained from KEGG rest API (https://www.kegg.jp/kegg/rest/keggapi.html) and GO annotations from the R software package http://org.hs.eg.db (version 3.1.0), to map genes into a background set. Then, the R software package clusterProfiler (Version 3.14.3) was used for enrichment analysis. We set the minimum gene set to 5 and the maximum gene set to 5000; *P* < 0.05 and FDR < 0.05 were considered to be statistically significant.

### 2.5. PPI Analysis

To identify more key genes related to DN, a PPI network of genes in the green module was established by the retrieval of interacting genes (STRING, http://string-db.org) [7]. A high confidence 0.9 was selected to design the PPI network. Then, the PPI network was visualized and the interactive relationships among interested genes were analyzed using the Cytoscape version 3.9.0 software. Genes were subsequently further identified by calculating the top 30 nodes ranked by degree method using cyto-hubba, a Cytoscape software plugin [“CytoHubba: identifying hub objects and subnetworks from complex interactome,” BMC Systems Biology].

### 2.6. External Validation

GSE99325 (18 DN samples and 6 controls) and GSE104954 (17 DN samples and 5 controls) were download from the GEO database to validate the hub gene-expression differences between the DN and control groups. A receiver-operating characteristic (ROC) curve was drawn to evaluate the efficiency of hub genes in the diagnosis of DN.

### 2.7. Clinical Features Analysis

The correlations between the expression of core genes and the GFR, proteinuria, SCR, and ACR values in DN patients were analyzed using the Nephroseq v5 online tool (http://v5.nephroseq.org/) by the Pearson correlation coefficient (cor). *P* < 0.05 was considered to be statistically significant. Insignificant results are not shown.

### 2.8. CMap Analysis

The CMap database (https://portals.broadinstitute.org/cmap) applies the whole genome-transcription system to comprehensively describe biological states, such as disease, physiology, and drug induction. The GSEA algorithm was used to extract and compare the gene-expression markers of these biological states so as to connect the drugs with the same (similar) or opposite functions, the diseases applicable to certain drugs, and the drug action pathways [8]. We used the genes in the green module to predict potential drugs that may ameliorate tubulointerstitial lesions in DN patients. Before CMap analysis, gene symbols were converted into probe identifiers according to the annotation information of the GPL96 chip, which was downloaded from the GEO database.

### 2.9. Statistical Analysis

Statistical analyses were handled with R (R Foundation for Statistical Computing, Vienna, Austria) and GraphPad Prism version 8.0 (GraphPad Software, Inc., La Jolla, CA, USA). An Unpaired *t-*test or the Mann–Whitney *U* test was used to evaluate the core genes' expression differences between the DN and control groups. ROC curves were established, and we calculated area under the ROC curve (AUC) values to evaluate the efficacy of core genes in diagnosing DN. The correlations between core gene expressions and GFR, proteinuria, SCR, and ACR values were assessed by the Pearson COR. All tests were two-tailed, and *P* < 0.05 indicated statistical significance.

## 3. Results

### 3.1. Identification of DEGs Specifically Involved in Tubulointerstitial Injury in DN

**Figure 1:**
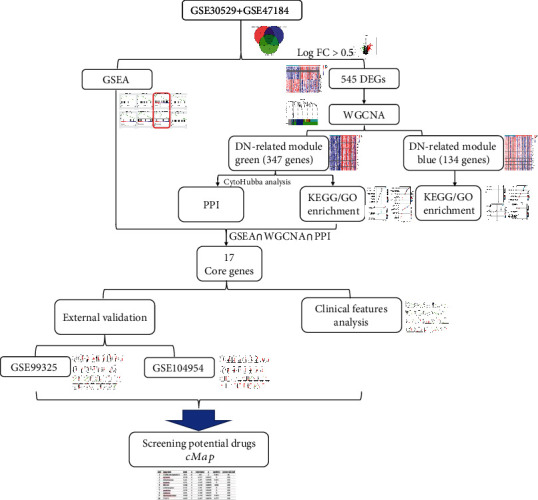
The flow chart of the study. Abbreviation: GSEA: gene set enrichment analysis; DEGs: differentially expressed genes; WGCNA: weighted gene coexpression network analysis; DN: diabetic nephropathy; PPI: protein-protein interaction.

Figure 1 shows the flow chart of the study. For screening DEGS, two GEO datasets, GSE30529 and GSE47184, were merged, incorporating a total of 28 DN samples and 18 controls (10 DN samples and 12 controls from GSE30529 and 18 DN samples and 6 controls from GSE47184, respectively). In addition, GSE99325 and GSE104954 were used for external validation, where GSE99325 included 18 DN samples and 6 tumor nephrectomy (TN) samples as control. GSE104954 included 17 DN samples and 5 TN samples as a control group.

**Figure 2:**
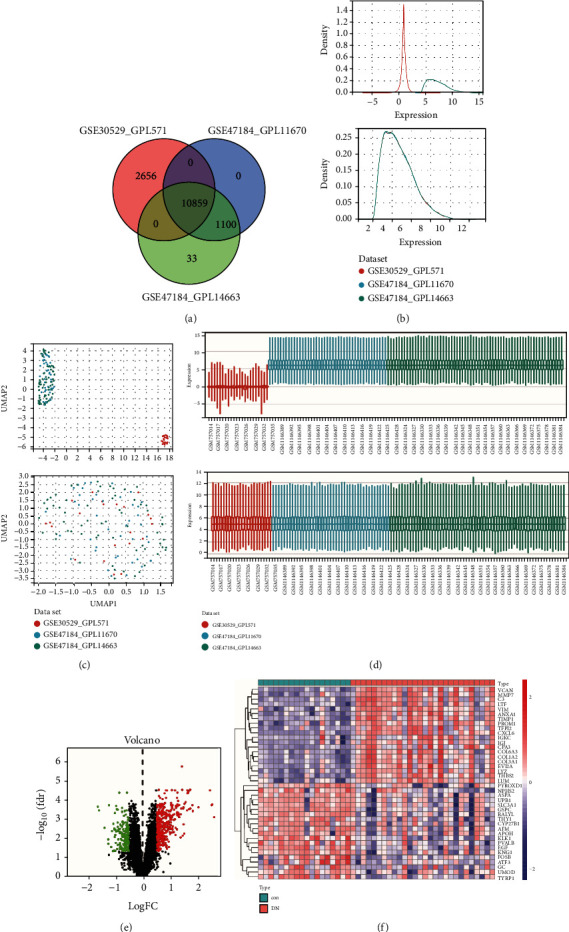
Identification of DEGs specifically implicated in tubulointerstitial injury in DN. (a) Venn diagram of merge of GSE30529-GPL571, GSE47184-GPL11670, and GSE47184-GPL14663, the two datasets showed an overlap of 10859 genes. (b–d) The density (b), UMAP (c), and boxplot (d) figure before or after removing batch. (e) Volcano plot analysis identifies DEGs, Log2FC > 0.5, and adj.*P* < 0.05. (f) Heat map of the top 20 upregulated and top 20 downregulated DEGs. Red: upregulation; blue: downregulation.

In this study, a total of 10859 genes were included after merging GSE30529 and GSE47184 (Figure 2(a)). Before removing the batch effect, the sample distribution of each dataset was observed to be quite different, suggesting that there was a batch effect. After removing the batch effect, the data distribution among each dataset tended to be consistent, with the median on the same line, and the mean and variance values also being similar to one another (Figures 2(b) and 2(c)). The UMAP diagram also showed that the batch effect was better removed (Figure 2(d)). After batch normalization, 545 DEGs were filtered out by the limma R package (adjusted *P* < 0.05 and |log2FC| > 0.5), in which 375 genes were upregulated, and 170 were downregulated (Figure 2(e)). Among them, the top 20 upregulated and the top 20 downregulated genes were exhibited on the heat map (Figure 2(f)). The comparison results before and after batch removal in GSE99325 and GSE104954 and the complete lists of 545 DEGs are presented in Figure S1 and Table S1, respectively (Figure S1, Table S1).

### 3.2. WGCNA and PPI Network Analysis

**Figure 3:**
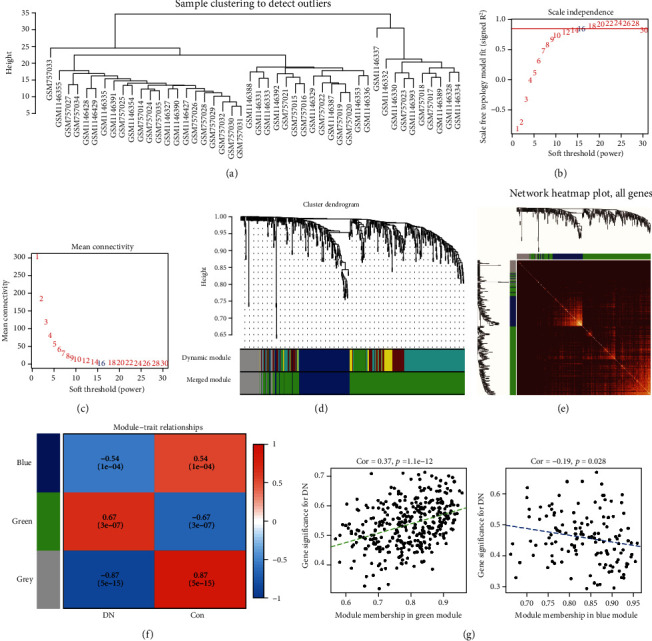
Identification of modules specially correlated with DN by WGCNA. (a) Sample dendrogram; (b) analysis of different soft-thresholding values from 1 to 30. (c) Evaluation of mean connectivity for each *β* value. *β* = 16 was selected. (d) Dendrogram of all DEGs clustered based on the dissimilarity measure (1-TOM). The original (upper bar) and merged modules (lower bar) are, respectively, shown in the two-colored bars below. (e) Network heat map plot of all the DEGs. Each row and column of the heat map belong to a single gene. The colors from red to progressive yellow represent a low to high adjacencies. (f) Module-trait relationships heat map, namely, correlation heat map of each module with clinical phenotype. (g) The relationship between the module membership and gene significance in the green (left) and blue (right) module.

**Figure 4:**
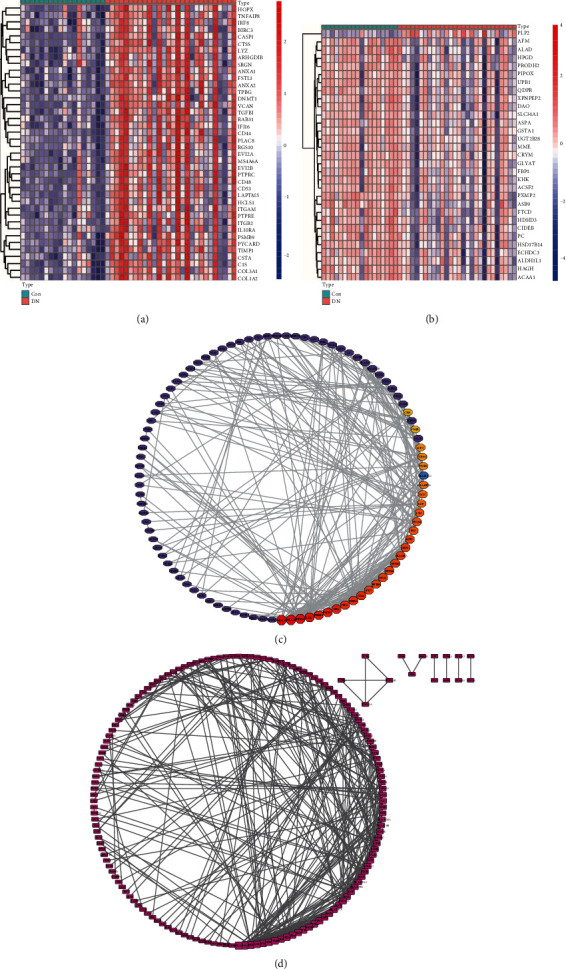
The heat map of hub genes selected by WGCNA and PPI network visualization of the coexpressed module. (a) The heat map of hub genes in the green module by WGCNA; (b) the heat map of hub genes in the blue module by WGCNA; (c) PPI network of all the genes in the green module based on STRING database; (d) PPI network of genes identified by calculating the top 30 nodes with neighbors and expanded ranked by degree method in “cyto-hubba,” a plug-in of Cytoscape software. Red: upregulation; blue: downregulation.

To identify the correlation between gene modules and the DN phenotype, WGCNA was performed based on DEGs of tubulointerstitial samples. No outlier among the samples existed based on sample clustering, and *β* = 16 (scale − free *R*^2^ = 0.85) was chosen as the soft-threshold power to conduct a scale-free gene coexpression network with complete module characteristics (Figure 3(a)). Modules were obtained by dynamic tree cutting and then were merged using following parameters: *β* = 16, minModuleSize = 20, deepSplit = 2; mergeCutHeight = 0.25. Namely, after generated by dynamic tree cut, modules were merged with a number of genes < 20 and the cutting height of 0.25. Then, three modules for the DEGs were obtained by WGCNA, including green, blue, and grey modules (Figures 3(b)–3(d)). The grey module was an unintentional module. Table S2 shows the numbers of genes in each module (Table S2). The adjacencies among genes and the module division consistency are exhibited in a heat map, which revealed a higher correlation among most of the genes in the same module (Figure 3(e)). Pearson's test was used to analyze the COR between the module eigengenes (MEs) and clinical traits. The association between individual genes and clinical traits was defined as the gene significance (GS), while the relationship between gene-expression values and MEs in certain module was denoted as the module membership (MM). The analysis between modules and clinical traits revealed that the green module was positively correlative with DN, with COR = 0.67 and *P* = 3*e* − 07, and the blue module was negatively correlative with DN, with COR = 0.54 and *P* = 1*e* − 04 (Figure 3(f)). Additionally, the outcomes of the GS vs. MM scatterplot in the green module showed a positively significant correlation (COR = 0.37, *P* = 1.1 − *e*12), while the blue module had a weak negatively significant relevance between the GS and MM (COR = −0.19, *P* = 0.028) (Figure 3(g)). Otherwise, used the cut-off criteria (|MM| > 0.9 and |GS| > 0.1). Forty genes in the green module and 31 genes in the blue module with high connectivity were considered to be hub genes and are shown in a heat map, respectively (Figures 4(a) and 4(b)).

A PPI network of the green module genes was developed based on the STRING database (Figure 4(c)). To further analyze module genes, a PPI subnetwork was developed by identifying the top 30 nodes with neighbors and expanded ranked by degree method in cyto-hubba, Cytoscape software plugin. A total of 95 genes were screened (Figure 4(d), Table S3).

### 3.3. KEGG and GO Enrichment Analyses of Green and Blue Module Genes

**Figure 5:**
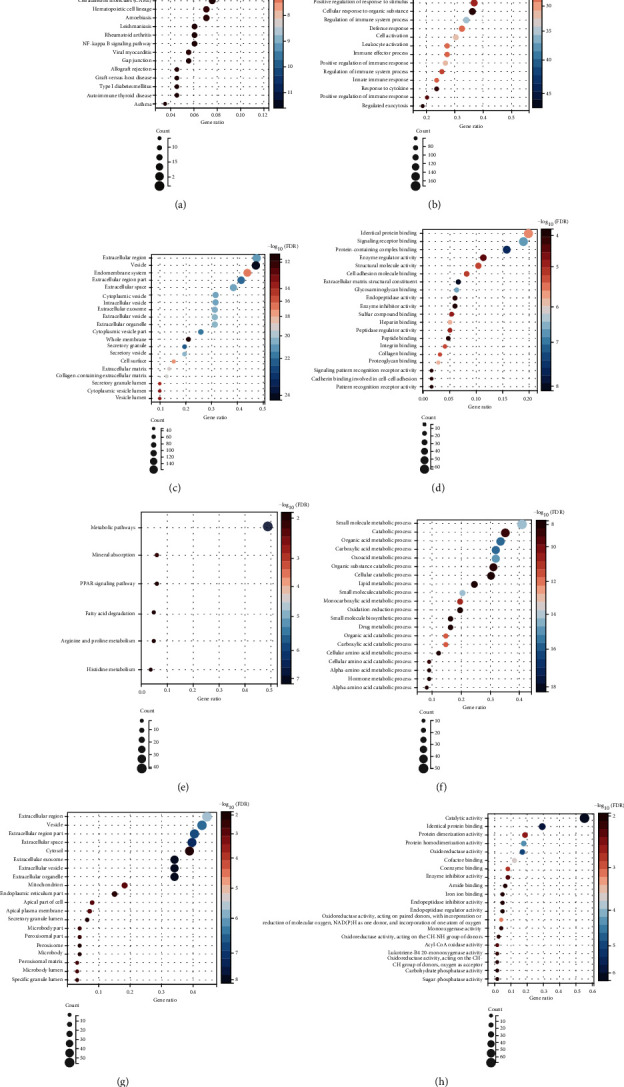
KEGG pathway and GO function enrichment analysis of genes assigned in the green and blue module. (a–d) The top 20 most statistically significant terms of KEGG (a), BP (b), CC (c), and MF (d) analysis of genes assigned in the green module; (e) the statistically significant terms of KEGG analysis of genes assigned in the blue module; (f–h) the top 20 most statistically significant terms of BP (b), CC (c), and MF (D) analysis of genes assigned in the blue module. The *x*-axis represents GeneRatio and *y*-axis represents KEGG/GO terms. The size of circle represents gene count. Different color of circles represents different adjusted *P* value; FDR: false discovery rate; KEGG: Kyoto Encyclopedia of Genes and Genomes; GO: Gene Ontology; MF: molecular function; BP: biological process; CC: cellular component.

To analyze the interrelated functions and pathways of genes in the green and blue modules, GO biological process (BP), GO molecular function (MF), GO cellular component (CC) analyses, and KEGG analysis were performed. The results of green module gene enrichment revealed that phagosome (hsa04145; *P* = 2.49*E* − 12) was most significantly enriched for in the KEGG pathway, followed by complement and coagulation cascades (hsa04610; *P* = 6.94*E* − 11), Staphylococcus aureus infection (hsa05150; *P* = 1.63*E* − 10), and so on (Figure 5(a) and Table S4). As for the BP, MF, and CC analyses, the outcomes are shown in Figures 5(b)–5(d). For the blue module, KEGG pathway enrichment analysis of blue module genes showed that metabolic pathways (hsa00140; *P* = 1.27*e* − 10), mineral absorption (hsa04978; *P* = 0.0004), and fatty acid degradation (hsa00071; *P* = 0.0012) were significantly enriched, as shown in Figure 5(e). Furthermore, the results of the BP, MF, and CC analyses are shown in Figures 5(f)–5(h).

### 3.4. Core Genes Related to Gene Tubulointerstitial Injury in DN Samples

**Figure 6:**
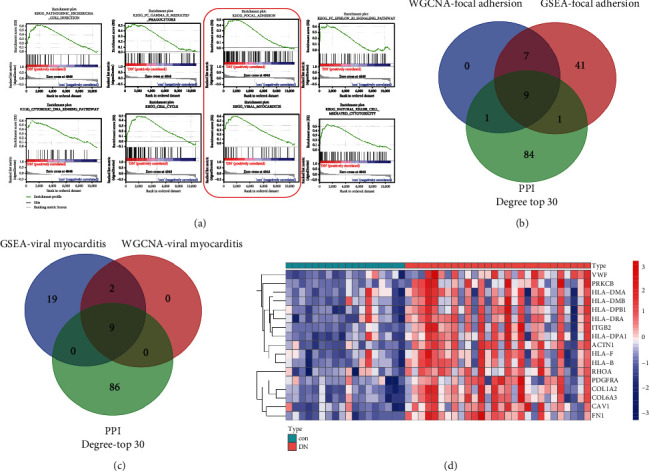
GSEA analysis and identified core gene related to the renal tubulointerstitial injury. GSEA plot showing most enriched gene sets from c2.cp.kegg.v7.4.symbols.gmt in the DN group. (a) The top 8 significant enriched gene set positively correlated with the DN group was KEGG_PATHOGENIC_ESCHERICHIA_COLI_INFECTION (ES = 0.631, NES = 1.573, *P* = 0.028, FDR = 0.237), KEGG_FC_GAMMA_R_MEDIATED_PHAGOCY-TOSIS (ES = 0.514, NES = 1.572, *P* = 0.029, FDR = 0.213), KEGG_FOCAL_ADHESION (ES = 0.489, NES = 1.563, *P* = 0.019, FDR = 0.207), KEGG_FC_EPSILON_RI_ SIGNALING_PATHWAY (ES = 0.433, NES = 1.552, *P* = 0.033, FDR = 0.211), KEGG_CYTOSOLIC_DNA_SENSING_PATHWAY (ES = 0.630, NES = 1.555, *P* = 0.016, FDR = 0.202), KEGG_CELL_CYCLE (ES = 0.508, NES = 1.542, *P* = 0.029, FDR = 0.213) (ES = 0.514, NES = 1.572, *P* = 0.029, FDR = 0.213) (ES = 0.514, NES = 1.572, *P* = 0.037, FDR = 0.181), KEGG_VIRAL_MYOCARDITIS (ES = 0.618, NES = 1.546, *P* = 0.047, FDR = 0.189), and KEGG_NATURAL_KILLER_CELL_MEDIATED_ CYTOTOXICITY (ES = 0.546, NES = 1.540, *P* = 0.033, FDR = 0.174). (b) Venn diagram of genes in WGCNA-focal adhesion pathway, GSEA-focal adhesion pathway, and the related genes of the top 30 nodes with neighbors and expanded ranked by degree method in “cyto-hubba” of PPI network. (c) Venn diagram of genes in WGCNA-viral myocarditis pathway, GSEA-viral myocarditis pathway, and the related genes of the top 30 nodes with neighbors and expanded ranked by degree method in “cyto-hubba” of PPI network. (d) The heat map of final 17 core genes in GSE30529-47184. WGCNA-focal adhesion: genes in focal adhesion pathway by WGCNA analysis; GSEA-focal adhesion: genes in focal adhesion pathway by GSEA KEGG analysis; WGCNA-viral myocarditis: genes in viral myocarditis pathway by WGCNA analysis; GSEA-viral myocarditis: genes in viral myocarditis pathway by GSEA KEGG analysis; PPI-degree top 30: the related genes of the top 30 nodes with neighbors and expanded ranked by degree method in “cyto-hubba” of PPI network; red: upregulation; blue: downregulation.

To identify KEGG signaling pathways enriched in the DN phenotype, GSEA was employed based on merged data from GSE30529 and GSE47184 and revealed significant differences (*P* < 0.05, FDR < 0.25) in enrichment using an annotated gene set (c2.cp.kegg.v7.2.symbols). As shown in Figure 6(a), the top 8 significantly enriched gene sets in positively correlated with the DN group were as follows: KEGG_PATHOGENIC_ESCHERICHIA_COLI_INFECTION (ES = 0.631, NES = 1.573, *P* = 0.028, FDR = 0.237), KEGG_FC_GAMMA_R_MEDIATED_ PHAGOCYTOSIS (ES = 0.514, NES = 1.572, *P* = 0.029, FDR = 0.213), KEGG_FOCAL_ADHESION (ES = 0.489, NES = 1.563, *P* = 0.019, FDR = 0.207), KEGG_FC_EPSILON_RI_SIGNALING_PATHWAY (ES = 0.433, NES = 1.552, *P* = 0.033, FDR = 0.211), KEGG_CYTOSOLIC_DNA_SENSING_PATHWAY (ES = 0.630, NES = 1.555, *P* = 0.016, FDR = 0.202), KEGG_CELL_CYCLE (ES = 0.508, NES = 1.542, *P* = 0.029, FDR = 0.213) (ES = 0.514, NES = 1.572, *P* = 0.029, FDR = 0.213) (ES = 0.514, NES = 1.572, *P* = 0.037, FDR = 0.181), KEGG_VIRAL_MYOCARDITIS (ES = 0.618, NES = 1.546, *P* = 0.047, FDR = 0.189), and KEGG_NATURAL_KILLER_CELL_MEDIATED_CYTOTOXICITY (ES = 0.546, NES = 1.540, *P* = 0.033, FDR = 0.174). From the GSEA results, the two pathways KEGG_FOCAL_ADHESION and KEGG_VIRAL_MYOCARDITIS were enriched, which were also confirmed by the results of KEGG analysis of green module genes in WGCNA. In order to lock core genes, the intersecting genes among the WGCNA focal adhesion pathway, GSEA focal adhesion pathway, and the related genes of the top 30 nodes with neighbors and expanded ranked by the degree method in cyto-hubba of the PPI network and the intersecting genes among WGCNA-viral myocarditis pathway, GSEA-viral myocarditis pathway, and the related genes of the top 30 nodes with neighbors and expanded ranked by degree method in cyto-hubba of the PPI network were analyzed (Figures 6(b) and 6(c)). Based on the Venn diagrams shown in Figures 6(b) and 6(c), upon synthesizing the results of the intersection, the final 17 core genes (ACTN1, CAV1, PRKCB, PDGFRA, COL1A2, COL6A3, RHOA, VWF, FN1, HLA-F, HLA-DPB1, ITGB2, HLA-DRA, HLA-DMA, HLA-DPA1, HLA-B, and HLA-DMB) related to tubulointerstitial lesions in DN were found (Figure 6(d)).

### 3.5. External Validation of the Expression and Diagnostic Capacity of Core Genes in the DN Group

**Figure 7:**
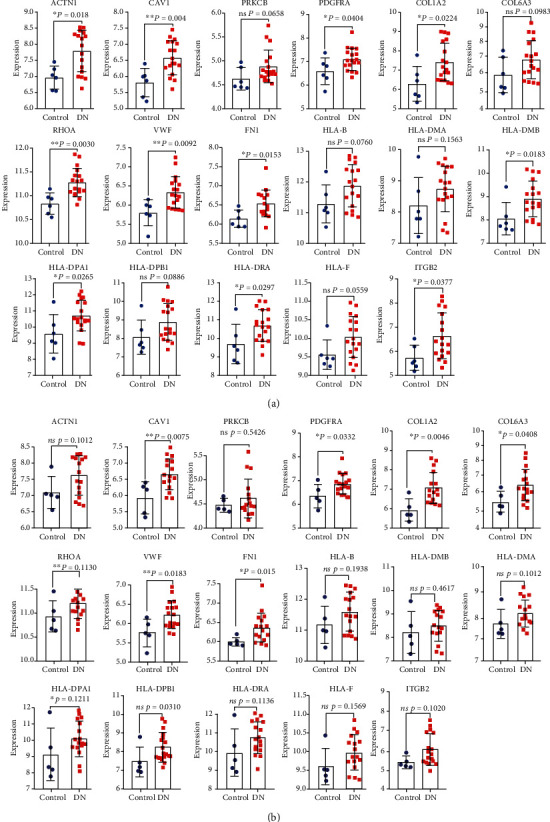
External validation on the different expression of core genes in renal tubulointerstitial tissues between DN patients and controls in dataset GSE99325 and GSE104954. (a) The different expression of core genes in renal tubulointerstitial tissues between DN patients and controls in dataset GSE99325; (b) the different expression of core genes in renal tubulointerstitial tissues between DN patients and controls in dataset GSE104954; analyzed by unpaired *t* test or Mann–Whitney *U* test ^∗^*P* < 0.05, ^∗∗^*P* < 0.01, ^∗∗∗^*P* < 0.001.

**Figure 8:**
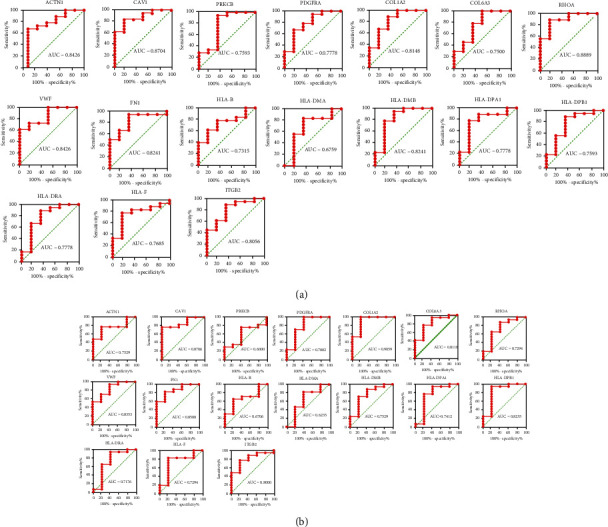
External validation on ROC curves of core genes in renal tubulointerstitial tissues in dataset GSE99325 and GSE104954. (a) ROC curves of core genes in renal tubulointerstitial tissues in dataset GSE99325; (b) ROC curves of core genes in renal tubulointerstitial tissues in dataset GSE104954. The AUCs were calculated.

As shown Figures 7 and 8, the two datasets of GSE99325 and GSE104954 were used for cross-validation. Five of the 17 core genes were identified from the expression level and diagnostic capacity in the DN group in validation datasets, including CAV1, PDGFRA, COL1A2, VWF, and FN1. In detail, the mRNA expression of CAV1 (*P* = 0.004 in GSE99325 and *P* = 0.0075 in GSE104954), PDGFRA (*P* = 0.0404 in GSE99325 and *P* = 0.0332 in GSE104954), COL1A2 (*P* = 0.0224 in GSE99325 and *P* = 0.0046 in GSE104954), VWF (*P* = 0.0092 in GSE99325 and *P* = 0.0183 in GSE104954), and FN1 (*P* = 0.0153 in GSE99325 and *P* = 0.015 in GSE104954) were significantly higher in the DN group than the control group in both GSE99325 and GSE104954 (Figure 7). The ROC curves showed that aside from HLA-DMA (AUC, 0.6759 in GSE99325 and AUC, 0.6235 in GSE104954), PRKCB (AUC, 0.6000 in GSE104954), and HLA-B (AUC, 0.6706 in GSE104954), the genes had relatively high AUCs (>0.7) in the diagnosis of DN, especially CAV1 (AUC, 0.8704 in GSE99325; AUC, 0.8706 in GSE104954), COL1A2 (AUC, 0.8148 in GSE99325; AUC, 0.9059 in GSE104954), VWF (AUC, 0.8426 in GSE99325; AUC, 0.8353 in GSE104954), FN1 (AUC, 0.8241 in GSE99325; AUC, 0.8588 in GSE104954), and ITGB2 (AUC, 0.8056 in GSE99325; AUC, 0.8000 in GSE104954) (Figure 8).

### 3.6. Clinical Validation on the Relationship between Core Genes and Kidney Function of Patients with DN

**Figure 9:**
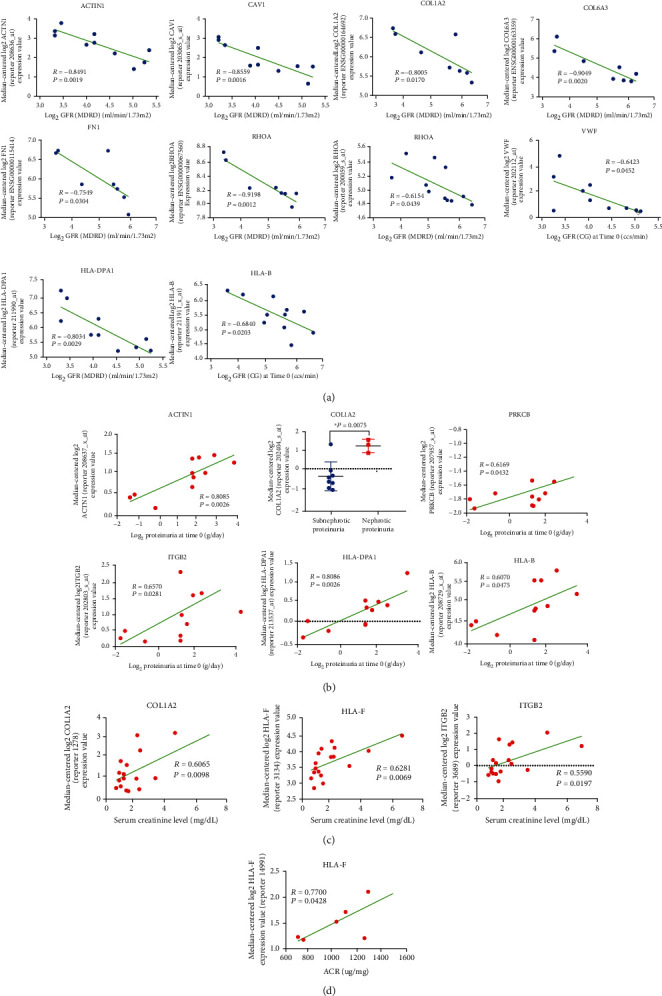
The correlation analysis between core genes renal tubulointerstium and clinical features in DN patients. (a) The significantly negative correlation between core genes in renal tubulointerstium and GFR in DN patients; (b) the significantly positive correlation between core genes in renal tubulointerstium and proteinuria in DN patients; (c) the significantly positive correlation between core genes in renal tubulointerstium and SCR in DN patients; (d) the significantly positive correlation between core genes in renal tubulointerstium and ACR in DN patients; DN: diabetic nephropathy; GFR: glomerular filtration rate; MDRD: modification of diet in renal disease; CG: Cockcroft Gault; SCR: serum creatinine level; ACR: urine albumin creatinine ratio; Pearson correlation of log2 transformed mRNA levels of core genes and clinical features in DN patients, *P* < 0.05 was statistically significant.

To verify potential roles of core genes in tubulointerstitial injury in DN, Pearson's correlation analysis between core genes and kidney function features like GFR, proteinuria, SCR, and ACR were analyzed using Nephroseq v5. Notably, the mRNA expression levels of ACTN1, CAV1, COL1A2, COL6A3, FN1, RHOA, VWF, HLA-DPA1, and HLA-B in kidney tubules were negatively relevant to the GFR in DN patients (Figure 9(a)), indicating that these core genes may promote the development of DN. Besides, compared to the subnephrotic proteinuria group, COL1A2 mRNA expression was significantly higher in the nephrotic proteinuria group (*P* = 0.0075), and the mRNA expression levels of ACTN1, PRKCB, ITGB2, HLA-DPA1, and HLA-B in kidney tubules were positively correlated with proteinuria in patients with DN (Figure 9(b)). In addition, the mRNA expression levels of COL1A1, HLA-F, and ITGB2 in kidney tubules were positively correlated with SCR in DN patients, hinting that those genes may facilitate the progression of DN (Figure 9(c)). Finally, HLA-F had a positive relationship with ACR in DN patients (Figure 9(d)).

### 3.7. Identification of Potential Drugs to Prevent Diabetic Tubulointerstitial Injury by CMap

To explore potential drugs for application in the treatment of DN, CMap analysis was performed on line based on the upregulated and downregulated genes in the critical green module. As shown in Table 1, the top 10 agents that may reverse the DEGs of the critical green module in cell lines were estradiol, LY-294002, 5224221, procaine, bufexamac, metaraminol, zimeldine, morantel, Prestwick-692, and PNU-0230031.

## 4. Discussion

With the prevalence of diabetes worldwide, the incidence rate of DN, as the major microvascular complication of this disease, has also increased rapidly and has become the main cause of ESRD both in developed and developing countries [9, 10]. Although the pathogenesis of DN has been extensively studied in recent decades, there are still no effective treatments available for DN in clinic. Therefore, early detection and early intervention should be emphasized for DN. It remains of great clinical significance to further discover the prospective diagnostic biomarkers, pathophysiological mechanisms, and possible intervention targets of DN.

Previously, studies on the mechanism of DN mostly focused on glomerular injury, and the clinical indicators used to evaluate DN are mostly based on the changes in glomerular structure and function [11]. Notably, emerging evidence suggests that tubular changes contribute to the progression of renal pathologies in diabetic kidney disease, including interstitial fibrosis. Pathologically, the lesions of patients with DN include thickness of the glomerular and tubular basement membranes, mesangial expansion, nodular glomerular sclerosis, and tubulointerstitial fibrosis [12], summarily, glomerular lesions and tubulointerstitial changes [13]. Formerly, DN was generally considered a glomerular disease, but with the deepening of research, compared to glomerular injury, it is believed that renal tubular disease and renal interstitial fibrosis are more closely related to the progressive deterioration of renal function and could be used as relatively independent indices to evaluate and predict the progress of renal disease [14–16]. Additionally, renal tubular interstitial accounts for >90% of renal parenchyma and is responsible for the varieties in pivotal functions, and renal tubular interstitial injury plays a central role in the progression of DN [17, 18]. In the development of chronic renal disease, renal interstitial fibrosis is a key pathological change and a predominant pathological feature in DN with tubular atrophy, extracellular matrix accumulation, and myofibroblast expansion [13, 19], which can better reflect the degree and level of renal damage. In renal interstitial fibrosis, histopathological changes in kidney tubules may be an initial factor, which have been regarded as an important cause of albuminuria and proteinuria [18]. Hence, identifying the susceptible genes of renal tubular injury in DN patients is very meaningful in order to elucidate the origin of this disease and explore potential treatments.

As evidence accumulates worldwide, DN is now known as the product of multiple gene interactions, but the molecular mechanisms at play remain poorly understood considering the complexity of etiological differences [20]. Therefore, potential biomarkers for early diagnosis and therapeutic targets were in sore need. Traditionally, single ChIP data had low reliability because of large individual differences and high false-positive ratios. The purpose of our investigation was to the ascertain underlying pathways and central genes related to the diagnosis and pathogenesis of DN in two merged GEO datasets, GSE30529 and GSE47184. Based on screening DEGs between DN patients and controls with log2FC > 0.5, the WGCNA algorithm was further performed for functional modules associated with diabetic tubulointerstitial injury. Compared to traditional microarray-based analysis methods, WGCNA possesses a number of unique advantages, which are characterized by analyzing gene clusters (modules) rather than entire genes and their interactions.

In this study, a total of 10,859 genes were included after GSE30529 and GSE47814 were merged, and we then identified 545 DEGs between renal tubulointerstitial tissues of 28 DN samples and 18 controls from the GSE30529 and GSE47184 merged datasets. The following WGCNA analysis showed that the green module was most significantly associated with DN, and KEGG/GO analysis was performed to investigate these genes in the green module further to elucidate more regarding the fundamental pathogenesis. Based on the results of KEGG pathway enrichment of genes in green module, as shown in Figure 5(a) and Table S4, the top 20 pathways (phagosome, complement and coagulation cascades, Staphylococcus aureus infection, pertussis, hematopoietic cell lineage, amoebiasis, viral myocarditis, allograft rejection, leishmaniasis, graft-versus-host disease, type I diabetes mellitus, tuberculosis, rheumatoid arthritis, cell adhesion molecules (CAMs), influenza A, autoimmune thyroid disease, NF-kappa B signaling pathway, asthma, focal adhesion, and gap junction) were mapped. Some of the pathways present here were consistent with previous studies [21–24]; complement and coagulation cascades, Staphylococcus aureus infection, pertussis were also enriched in Xu et al.'s research [21]. While hematopoietic cell lineage, amoebiasis, NF-kappa B signaling pathway, complement and coagulation cascades, pertussis were also enriched after WGCNA analysis in Iup et al.'s study [25]. Phagosome, complement and coagulation cascades, Staphylococcus aureus infection, pertussis, viral myocarditis, allograft rejection, leishmaniasis, graft-versus-host disease, type I diabetes mellitus, tuberculosis, rheumatoid arthritis, cell adhesion molecules (CAMs), influenza A, autoimmune thyroid disease, asthma and focal adhesion were related to DN in Zeng et al.'s article [23]. Complement and coagulation cascades, hematopoietic cell lineage, cell adhesion molecules (CAMs), NF-kappa B signaling pathway, phagosome, focal adhesion were enriched in Cai et al.'s research [24]. All of the above findings suggest the reliability of KEGG enrichment. As for GO annotation in green module, there were predominantly enriched on immune-related processes, like immune system process, immune response, regulation of immune system process, regulation of immune response, and immune effector process, in the GO BP enrichment analysis (Figure 5(b)), indicating the exceptionally active immune process in diabetic tubulointerstitial injury. Although DN is not a conservative immune-mediated renal disease, there is growing evidence that immune system components involved in the progression of DN are exhibited in affected patients [26]. Many clinical studies had reported that the activation of T-cells [27] and the increase of immune complexes [28, 29] are associated with nephropathy progression in patients with DM. Moreover, GO CC and GO MF analysis were enriched for genes located in extracellular vesicles, including extracellular region, extracellular region part, extracellular space, extracellular exosome, and extracellular matrix structural constituent, indicating the extreme activation of profibrotic processes in patients with DN (Figures 5(c) and 5(d)), which was also recognized by other researchers [30–32].

GSEA is a kind of calculation used to determine whether a predefined set of genes shows statistically significant and consistent differences between two biological states. Intriguingly, both the KEGG pathway analysis of genes in green module based on the WGCNA algorithm and the GSEA-annotated KEGG gene set analysis were enriched in two identical pathways (focal adhesion and viral myocarditis), suggesting these critical role of the two pathways. Notably and uniquely, to lock the core genes associated with renal tubule injury in DN, a PPI network of genes in the green module was developed. We selected cross genes as core genes among WGCNA focal adhesion or viral myocarditis, GSEA focal adhesion or viral myocarditis, and the top 30 nodes with neighbors and expanded ranked by the degree method in cyto-hubba of the green module PPI network. Finally, a total of 17 genes distinguished as core genes, namely, *ACTN1*, *CAV1*, *PRKCB*, *PDGFRA*, *COL1A2*, *COL6A3*, *RHOA*, *VWF*, *FN1*, *HLA-F*, *HLA-DPB1*, *ITGB2*, *HLA-DRA*, *HLA-DMA*, *HLA-DPA1*, *HLA-B*, and *HLA-DMB*. *CAV1* [33], Caveolin-1, acts as a scaffolding protein within caveolar membranes [34] and is crucial to promote profibrotic signal transduction resulted from several known stimuli in DN, such as the most prominent factors hyperglycemia and angiotensin II, thus representing a novel and hopeful therapeutic option for DN [33]. *PRKCB*, protein kinase C beta type, is a kind of PKC isoforms. Langham et al. reported that *PRKCB* mRNA expression was upregulated expression and correlated closely with serum HbA (1c) in patients with DN [35]. *COL1A2*, collagen type I *α*2 chain, is closely positively related to the progression of renal fibrosis in DN [36]. Zeng et al. [23, 24] found that *COL6A3* may contribute to kidney injury in DN. Researchers also documented upregulated expressions of *RHOA* and *VWF* in DN tubule samples [37]. *FN1* was reported negatively related with GFR in patients with DN [38] and was upregulated in podocytes by mechanical stress [39]. *ITGB2* was identified as a hub gene from the complement cascade pathway and negatively correlated with GFR [21]. Ma et al. [40] determined that *HLA-DPA1* was a potential key gene related to the development of DKD involved in immune regulation. *HLA-B* was proven to be a member of the NF-kappaB module NFKB_IRFF_01, which, was activated in the inflammatory stress response of progressive DN and could be a potential target for the treatment of progressive renal diseases such as DN [41].

Few or no studies exist on the relationship between DN and core genes, such as *ACTN1*, *PDGFRA*, *HLA-F*, *HLA-DPB1*, *HLA-DRA*, *HLA-DMA*, and *HLA-DMB*. However, all of these genes were upregulated in renal tubulointerstitial tissues of patients with DN and may have an exacerbated role in the development of diabetic tubulointerstitial injury. *ACTN1*, actinin alpha 1, belongs to the spectrin gene superfamily, which is a diverse group of cytoskeletal proteins, including the *α* and *β* spectrins and dystrophins, of which *α*-actinins are major cytoskeletal proteins based on their critical role in cell adhesion and the organization of the cytoskeleton [42]. It is worth noting that cytoskeletal changes are observed in podocytes in diabetes [43]. One member of *α*-actinins, *α*-actinin-4, was highly expressed at the foot processes of the podocytes and in blood vessel walls in the normal kidneys [44], and its function mutations were associated with familial focal and segmental glomerulosclerosis [45]. It reveals that *ACTN1*, as an *α*-actinins, may induce renal tubule injury though cytoskeletal changes. *PDGFRA*, platelet-derived growth factor receptor alpha, which encodes a cell surface tyrosine kinase receptor, plays a crucial role in organ development, wound healing, and tumor progression. Song et al. [46] found that there was reinforced activation of the expression of *PDGFRA* and hedgehog signaling in adventitial cells of AVFs from patients with ESKD and CKD mice. *PDGFRA* was translocated and accumulated in early endosomes, followed by sonic hedgehog overexpression. *HLA-F*, which belongs to the human leukocyte antigen (HLA) class I heavy chain paralog family, is mainly localized in the endoplasmic reticulum and Golgi apparatus, differing from most other HLA heavy chains. It plays an important role in immune surveillance, immune tolerance, and inflammation. Additionally, both *HLA-DRA* and *HLA-DMA* belong to the HLA class II *α*-chain paralog family, while *HLA-DPB1* and *HLA-DMB* belong to the HLA class II *β*-chain paralog family, all of which anchor in the membrane and play a central role in the immune system by presenting peptides derived from extracellular proteins. Many researchers found a correlation for the *DPB1*, *DRA*, *DMA*, and *DMB* locus in patient susceptibility to type 1 diabetes [47–49], but there is lack of related reports about DN.

In the present study, after clinical cross and index validation from GSE99325, GSE104954, and the Nephroseq v5 platform, the expression of *ACTN1*, *CAV1*, *PDGFRA*, *COL1A2*, *COL6A3*, *RHOA*, *VWF*, *FN1*, *HLA-DPB1*, *ITGB2*, *HLA-DRA*, *HLA-DPA1*, and *HLA-DMB* were significantly upregulated in DN patients in at least one dataset, GSE99325 or GSE104954. As for the clinical feature analysis of core genes, it offered the positive results that the mRNA expressions of *ACTN1*, *CAV1*, *COL1A2*, *COL6A3*, *FN1*, *RHOA*, *VWF*, *HLA-DPA1*, and *HLA-B* in kidney tubules negatively correlated with GFR, and the mRNA expression of *ACTN1*, *COL1A2*, *PRKCB*, *ITGB2*, *HLA-DPA1*, and *HLA-B* in kidney tubules positively correlated with proteinuria; the mRNA expressions of *COL1A1*, *HLA-F*, and *ITGB2* in kidney tubules positively correlated with SCR; the *HLA-F* mRNA expressions level positively correlated with ACR in DN patients.

CMap, an online tool to analyze potential therapeutic drugs based on upregulated and downregulated genes, is used in many diseases. Estradiol, lists as the top medication, is a renoprotective drug. As early as 2005, Wells et al. [50] discovered that estradiol supplementation may be an effective method to reduce the occurrence and progression of diabetic kidney complications. They also found that 17*β*-estradiol replacement improved renal function and the pathology associated with DN [51]. Specifically, it reduced tubulointerstitial fibrosis by increasing matrix metalloproteinase activity [52] and attenuated DN by regulating the extracellular matrix and transforming growth factor- (TGF-) *β* protein expression and signaling [53] in DN. LY-294002, a pharmacological inhibitor of PI-3 kinase, inhibits the activation of the catalytic subunit (p110) of PI-3 kinase [54]. LY294002 could inhibit the expression of osteopontin, a secreted phosphoprotein involved in the progression of tubulointerstitial inflammation, which is stimulated by glucose in primary cultures of human renal proximal tubular epithelial cells [55]. Bufexamac, an aryl alkanoic acid derivative and a nonsteroidal anti-inflammatory agent for the topical treatment of eczema and other inflammatory skin diseases [56], is a specific inhibitor of the deacetylases of histone types IIB (HDAC6 and HDAC10 [57]). Liang et al. [58] proved that HDAC6, a kind of histone deacetylase (HDAC), downregulated the acetylation of *α*-tubulin, heightened motility, and restrained autophagy in podocytes dealing with AGE, which deteriorated the phenotype of DN, suggesting that HDAC6 is a prospective target for therapy in the early phase of DN. In addition, growing data suggest that the inhibition of HDAC can ameliorate clinical manifestations of diabetic kidney disease and phenotypes such as fibrosis, inflammation, cell death, and albuminuria [59–61]. Notably, there is no relevant experiment on the effects of bufexamac in the treatment of diabetic tubulointerstitial injury. Intriguingly, LY294002, which blocks the PI3K/Akt pathway, inhibited high glucose-induced epithelial–mesenchymal transition (EMT) in HK2 cells through reduced HDAC5 expression in a TGF-*β*1-dependent way. The intake of TSA, another HDAC inhibitor, also reduced HDAC5 expression and then suppressed EMT in the kidneys of diabetic mice. It is meaningful and promising to explore single or combined application of LY294002 and bufexamac to treat diabetic tubule damage. Currently, there are only 309 gene-expression maps of known compounds in the CMap database, which cannot cover all the requirements for comparing all drug gene-expression maps, and there is the problem of “comparison loss” of action mechanism. Therefore, the effects of LY294002 and bufexamac in the treatment of diabetic kidney injury need to be further verified by in vitro and in vivo experiments.

## 5. Conclusions

Conclusively, the present study sought to identify core biomarkers implicated in diabetic tubulointerstitial injury. A total of 17 core genes was screened out and locked, which may be potential targets for the diagnosis and therapy of DN in the future. Besides, two prospective small compounds were also found that may be potential therapeutic drugs in diabetic kidney disease. However, some limitations in the article existed as well. Clinical validation of the diagnostic performance of core genes should be further pursued, and basic studies are needed to validate the focal adhesion and viral myocarditis pathways related to diabetic tubule lesions.

## Figures and Tables

**Table 1:** The potential drugs analyzed by CMap analysis to reverse altered expression of genes in the green module.

Rank	cmap name and cell line	Mean	*n*	Enrichment	*P*	Cell
1	Estradiol	-0.429	8	-0.649	0.00086	PC3
2	LY-294002	-0.245	13	-0.522	0.0011	HL60
3	5224221	-0.652	2	-0.967	0.00239	MCF7
4	Procaine	-0.65	2	-0.961	0.0033	PC3
5	Bufexamac	-0.64	2	-0.961	0.00334	MCF7
6	Metaraminol	-0.666	2	-0.959	0.00364	PC3
7	Zimeldine	-0.635	2	-0.958	0.0038	PC3
8	Morantel	-0.663	2	-0.946	0.0063	PC3
9	Prestwick-692	-0.612	2	-0.944	0.0069	MCF7
10	PNU-0230031	-0.387	4	-0.737	0.00953	MCF7

## Data Availability

The datasets generated during and/or analyzed during the current study are presented in the main file. Additional data are available from the corresponding author on reasonable request.

## Supplementary Materials

Supplementary Materials Figure S1: the boxplot figure before or after removing batch in GSE99325 and GSE104954. Table S1: 545 DEGS. Table S2: the number of genes and the identified hub genes in each coexpression module. Table S3 related genes the top 30 nodes with neighbors and expanded ranked by degree method in “cyto-hubba” of PPI network. Table S4: KEGG analysis of green module genes.
