# Supplementary material for: Integrated Analysis of Multiple Microarray Studies to Identify Core Gene-Expression Signatures Involved in Tubulointerstitial Injury in Diabetic Nephropathy
Source: Biomed Res Int. 2022 May 10;2022:9554658. doi: 10.1155/2022/9554658 (PMC9113875; doi:10.1155/2022/9554658)
Supplement: Supplementary Materials — Figure S1: the boxplot figure before or after removing batch in GSE99325 and GSE104954. Table S1: 545 DEGS. Table S2: the number of genes and the identified hub genes in each coexpression module. Table S3 related genes the top 30 nodes with neighbors and expanded ranked by degree method in “cyto-hubba” of PPI network. Table S4: KEGG analysis of green module genes. [file 9554658.f1.zip › Table S1.docx]

Table S1 545 DEGS

| Gene | conMean | treatMean | logFC | pValue | fdr |
| --- | --- | --- | --- | --- | --- |
| LTF | 5.9313 | 8.519411 | 2.58811 | 2.06E-05 | 0.000817 |
| IGJ | 4.733078 | 7.249655 | 2.516577 | 7.87E-07 | 0.00018 |
| LYZ | 4.468634 | 6.966805 | 2.498171 | 1.26E-06 | 0.0002 |
| CXCL6 | 3.498976 | 5.936466 | 2.43749 | 3.02E-08 | 3.30E-05 |
| MMP7 | 7.382243 | 9.408969 | 2.026726 | 2.32E-06 | 0.000256 |
| C3 | 6.269549 | 8.089453 | 1.819905 | 5.49E-06 | 0.000399 |
| PROM1 | 6.139293 | 7.945009 | 1.805716 | 1.26E-06 | 0.0002 |
| TIMP1 | 6.305089 | 8.037996 | 1.732907 | 3.11E-06 | 0.000296 |
| ANXA1 | 5.271233 | 7.002699 | 1.731466 | 1.60E-08 | 3.15E-05 |
| VCAN | 3.672878 | 5.386218 | 1.713341 | 1.60E-08 | 3.15E-05 |
| COL3A1 | 5.400572 | 7.088449 | 1.687878 | 3.59E-06 | 0.000323 |
| CPA3 | 4.183163 | 5.845261 | 1.662098 | 5.51E-08 | 3.86E-05 |
| LUM | 6.248609 | 7.891528 | 1.642919 | 8.12E-08 | 4.42E-05 |
| THBS2 | 5.137108 | 6.768616 | 1.631508 | 9.81E-08 | 4.81E-05 |
| COL1A2 | 4.768029 | 6.387685 | 1.619657 | 2.45E-08 | 3.30E-05 |
| COL6A3 | 4.56379 | 6.17605 | 1.61226 | 4.77E-06 | 0.000372 |
| VIM | 8.141402 | 9.701668 | 1.560265 | 4.07E-07 | 0.000138 |
| EVI2A | 3.739942 | 5.256354 | 1.516413 | 5.68E-07 | 0.000169 |
| IGKC | 8.285084 | 9.792508 | 1.507424 | 0.000499 | 0.005883 |
| TFPI2 | 4.505058 | 5.988015 | 1.482956 | 7.64E-05 | 0.001921 |
| LY96 | 3.634192 | 5.115964 | 1.481772 | 2.32E-06 | 0.000256 |
| TNC | 4.882027 | 6.353038 | 1.47101 | 3.11E-06 | 0.000296 |
| SERPINA3 | 6.035842 | 7.484412 | 1.448571 | 0.000728 | 0.007482 |
| CX3CR1 | 4.58253 | 6.015145 | 1.432616 | 1.03E-08 | 3.15E-05 |
| AGR2 | 4.032732 | 5.456426 | 1.423693 | 1.93E-10 | 1.89E-06 |
| TAC1 | 3.245716 | 4.640602 | 1.394886 | 3.11E-06 | 0.000296 |
| HOPX | 4.461793 | 5.843357 | 1.381564 | 1.08E-05 | 0.00057 |
| CCL19 | 4.205395 | 5.582004 | 1.376609 | 0.001047 | 0.009425 |
| KRT19 | 5.866682 | 7.192197 | 1.325515 | 2.06E-05 | 0.000817 |
| MS4A4A | 3.718656 | 5.040387 | 1.321731 | 1.08E-06 | 0.000196 |
| C1S | 5.820531 | 7.141443 | 1.320912 | 1.23E-05 | 0.000602 |
| TRIM22 | 5.837582 | 7.132389 | 1.294807 | 8.28E-06 | 0.000504 |
| PLAC8 | 3.574832 | 4.869046 | 1.294214 | 1.60E-05 | 0.000693 |
| IFI16 | 4.522574 | 5.804998 | 1.282424 | 3.44E-07 | 0.00012 |
| LAPTM5 | 5.60002 | 6.869026 | 1.269006 | 1.72E-06 | 0.000222 |
| PLK2 | 3.643248 | 4.904548 | 1.2613 | 6.70E-08 | 4.38E-05 |
| CD53 | 5.964119 | 7.222104 | 1.257985 | 7.87E-07 | 0.00018 |
| CD48 | 4.964129 | 6.213321 | 1.249191 | 7.23E-06 | 0.000467 |
| ANXA2 | 8.421317 | 9.669665 | 1.248348 | 1.26E-06 | 0.0002 |
| HLA-DPA1 | 7.574721 | 8.822602 | 1.247881 | 1.40E-05 | 0.000629 |
| EVI2B | 3.428256 | 4.673845 | 1.245588 | 2.04E-07 | 8.33E-05 |
| SLPI | 6.099461 | 7.342898 | 1.243437 | 0.000225 | 0.00356 |
| TGFBI | 5.787829 | 7.025015 | 1.237185 | 8.28E-06 | 0.000504 |
| RNASE6 | 4.360446 | 5.572015 | 1.211569 | 2.69E-06 | 0.000278 |
| POSTN | 4.434017 | 5.629252 | 1.195235 | 0.000164 | 0.002938 |
| QPCT | 3.88282 | 5.076525 | 1.193705 | 1.47E-06 | 0.000219 |
| C1QB | 5.049881 | 6.235981 | 1.1861 | 0.000663 | 0.006978 |
| FSTL1 | 6.861006 | 8.031708 | 1.170702 | 3.70E-08 | 3.30E-05 |
| PYCARD | 3.953604 | 5.122444 | 1.16884 | 2.98E-05 | 0.001049 |
| MS4A6A | 5.225442 | 6.370765 | 1.145323 | 1.08E-05 | 0.00057 |
| TNFAIP8 | 5.275752 | 6.412799 | 1.137047 | 4.14E-06 | 0.000345 |
| TYROBP | 5.327888 | 6.453012 | 1.125123 | 0.000373 | 0.004906 |
| FCER1A | 3.565125 | 4.688435 | 1.123309 | 3.37E-05 | 0.00114 |
| IFITM1 | 7.056124 | 8.155269 | 1.099145 | 0.000373 | 0.004906 |
| HCLS1 | 4.62633 | 5.72069 | 1.09436 | 1.40E-05 | 0.000629 |
| GZMA | 3.911756 | 5.002968 | 1.091211 | 0.000119 | 0.002464 |
| TMSB10 | 9.079329 | 10.15785 | 1.078526 | 1.26E-06 | 0.0002 |
| ADH1B | 5.651208 | 6.727218 | 1.07601 | 0.000225 | 0.00356 |
| TRBC1 | 5.852637 | 6.922768 | 1.070131 | 9.23E-07 | 0.000196 |
| LOXL1 | 4.232457 | 5.301086 | 1.068629 | 2.69E-06 | 0.000278 |
| HLA-DPB1 | 7.713837 | 8.780754 | 1.066916 | 0.000183 | 0.003155 |
| COL15A1 | 4.018577 | 5.075487 | 1.05691 | 2.00E-06 | 0.000239 |
| ANXA3 | 4.89393 | 5.949457 | 1.055526 | 2.06E-05 | 0.000817 |
| CLEC2B | 4.915535 | 5.970804 | 1.05527 | 1.99E-08 | 3.25E-05 |
| TSPAN13 | 6.019693 | 7.074491 | 1.054798 | 1.72E-06 | 0.000222 |
| HLA-DRA | 7.824225 | 8.87464 | 1.050415 | 0.000411 | 0.005237 |
| ITGB2 | 4.511697 | 5.552036 | 1.04034 | 1.60E-05 | 0.000693 |
| PSMB9 | 5.261183 | 6.301261 | 1.040078 | 2.98E-05 | 0.001049 |
| LGALS1 | 6.54675 | 7.585652 | 1.038901 | 0.000183 | 0.003155 |
| ARL4C | 4.566817 | 5.601105 | 1.034289 | 4.14E-06 | 0.000345 |
| MARCKSL1 | 5.743334 | 6.769512 | 1.026178 | 4.82E-07 | 0.000152 |
| CASP1 | 4.027496 | 5.053562 | 1.026066 | 1.08E-06 | 0.000196 |
| ARHGDIB | 5.834975 | 6.852795 | 1.01782 | 5.41E-05 | 0.001539 |
| SRGN | 6.051287 | 7.067929 | 1.016642 | 9.56E-05 | 0.00216 |
| FHL2 | 5.664533 | 6.678683 | 1.01415 | 3.37E-05 | 0.00114 |
| MTHFD2 | 4.18055 | 5.188377 | 1.007827 | 0.000203 | 0.003322 |
| PTPRC | 3.856816 | 4.863571 | 1.006755 | 5.49E-06 | 0.000399 |
| TPBG | 5.239917 | 6.237935 | 0.998018 | 2.64E-05 | 0.000952 |
| MGP | 7.879642 | 8.877367 | 0.997725 | 0.000183 | 0.003155 |
| CLU | 7.005908 | 8.000931 | 0.995024 | 0.000107 | 0.002316 |
| SERPINF1 | 5.653758 | 6.641265 | 0.987506 | 0.000133 | 0.002614 |
| RARRES1 | 4.341767 | 5.329249 | 0.987482 | 0.000549 | 0.006219 |
| WFDC2 | 6.402647 | 7.384955 | 0.982308 | 0.000203 | 0.003322 |
| MNDA | 3.474124 | 4.452404 | 0.978281 | 1.81E-05 | 0.000757 |
| CSTA | 3.593957 | 4.569626 | 0.975669 | 0.000373 | 0.004906 |
| CAV1 | 4.634091 | 5.600467 | 0.966376 | 1.23E-05 | 0.000602 |
| TUBA1A | 7.724359 | 8.68937 | 0.965011 | 1.08E-05 | 0.00057 |
| BASP1 | 5.226579 | 6.184816 | 0.958237 | 0.000164 | 0.002938 |
| FGL2 | 6.397729 | 7.35586 | 0.958131 | 1.40E-05 | 0.000629 |
| CHST15 | 4.675911 | 5.625886 | 0.949975 | 5.49E-06 | 0.000399 |
| C1QA | 5.050508 | 5.99955 | 0.949043 | 0.006258 | 0.029505 |
| IFITM2 | 7.958388 | 8.904457 | 0.946069 | 7.64E-05 | 0.001921 |
| HTR2B | 3.320883 | 4.263957 | 0.943074 | 5.49E-06 | 0.000399 |
| CFH | 4.518152 | 5.459417 | 0.941265 | 5.41E-05 | 0.001539 |
| CCDC109B | 4.175349 | 5.113589 | 0.938241 | 4.28E-05 | 0.001355 |
| CD3D | 4.224527 | 5.162086 | 0.937559 | 0.00227 | 0.015466 |
| COL4A1 | 6.649197 | 7.575803 | 0.926606 | 1.23E-05 | 0.000602 |
| ARPC1B | 6.407484 | 7.331626 | 0.924142 | 4.82E-05 | 0.001441 |
| IRF8 | 5.361004 | 6.279058 | 0.918054 | 0.000133 | 0.002614 |
| CD163 | 5.303231 | 6.216018 | 0.912786 | 0.000249 | 0.003769 |
| SAMSN1 | 3.213845 | 4.121047 | 0.907202 | 0.000225 | 0.00356 |
| IGFBP6 | 5.57947 | 6.485519 | 0.906049 | 0.000148 | 0.002767 |
| A2M | 7.633799 | 8.536277 | 0.902477 | 8.55E-05 | 0.00205 |
| ACTN1 | 5.439955 | 6.340771 | 0.900816 | 1.23E-05 | 0.000602 |
| CRIP1 | 5.018579 | 5.910753 | 0.892174 | 6.82E-05 | 0.001803 |
| CTSS | 4.11309 | 5.00474 | 0.89165 | 1.23E-05 | 0.000602 |
| PLSCR1 | 6.893088 | 7.783204 | 0.890116 | 1.60E-05 | 0.000693 |
| C7 | 7.409719 | 8.29841 | 0.888691 | 0.001488 | 0.011832 |
| RCN1 | 5.796048 | 6.679541 | 0.883493 | 1.40E-05 | 0.000629 |
| SLC2A10 | 3.468277 | 4.346316 | 0.87804 | 4.52E-08 | 3.41E-05 |
| SELL | 3.886011 | 4.762393 | 0.876382 | 6.82E-05 | 0.001803 |
| RND3 | 5.736665 | 6.612915 | 0.876249 | 0.000337 | 0.004604 |
| LCN2 | 4.448777 | 5.321275 | 0.872498 | 0.008356 | 0.036211 |
| FKBP11 | 5.688083 | 6.559801 | 0.871717 | 4.82E-05 | 0.001441 |
| PDLIM1 | 7.401406 | 8.270563 | 0.869158 | 4.77E-06 | 0.000372 |
| NPY1R | 6.359928 | 7.219237 | 0.859309 | 0.000874 | 0.008417 |
| GJA1 | 5.11007 | 5.967573 | 0.857503 | 0.004638 | 0.024216 |
| S100A4 | 6.05001 | 6.906313 | 0.856302 | 0.000148 | 0.002767 |
| GZMK | 4.782991 | 5.63579 | 0.852799 | 0.006258 | 0.029505 |
| PLP2 | 5.71941 | 6.570455 | 0.851046 | 8.28E-06 | 0.000504 |
| GMFG | 5.056275 | 5.905743 | 0.849468 | 0.000305 | 0.004322 |
| TNFRSF17 | 3.048023 | 3.894364 | 0.846341 | 2.06E-05 | 0.000817 |
| C1R | 5.898216 | 6.743474 | 0.845258 | 0.000874 | 0.008417 |
| FN1 | 4.701989 | 5.545545 | 0.843556 | 2.04E-07 | 8.33E-05 |
| SRPX | 3.688535 | 4.529127 | 0.840593 | 2.64E-05 | 0.000952 |
| TMEM45A | 3.204186 | 4.044296 | 0.840111 | 3.70E-08 | 3.30E-05 |
| IFI44L | 4.256125 | 5.095023 | 0.838898 | 0.000337 | 0.004604 |
| SPARC | 6.353739 | 7.191206 | 0.837466 | 1.40E-05 | 0.000629 |
| IFITM3 | 7.949327 | 8.785321 | 0.835994 | 0.000411 | 0.005237 |
| LHFP | 5.403255 | 6.238043 | 0.834788 | 0.000119 | 0.002464 |
| IFI27 | 6.285049 | 7.115267 | 0.830218 | 0.004409 | 0.023666 |
| CCL5 | 3.913235 | 4.735517 | 0.822282 | 0.003141 | 0.01888 |
| IL10RA | 4.251311 | 5.073381 | 0.82207 | 0.002899 | 0.017966 |
| RASSF2 | 3.714179 | 4.534505 | 0.820327 | 6.08E-05 | 0.00167 |
| SERPING1 | 6.577623 | 7.394031 | 0.816408 | 0.001622 | 0.012538 |
| LY86 | 5.129691 | 5.93904 | 0.809349 | 2.33E-05 | 0.00089 |
| BST2 | 5.55846 | 6.366217 | 0.807758 | 0.001365 | 0.011157 |
| RGS10 | 4.750457 | 5.555851 | 0.805394 | 9.56E-05 | 0.00216 |
| RAB31 | 5.020027 | 5.824143 | 0.804116 | 2.06E-05 | 0.000817 |
| MYOF | 5.732019 | 6.535607 | 0.803588 | 4.82E-07 | 0.000152 |
| CD69 | 3.06355 | 3.865975 | 0.802424 | 0.000865 | 0.008417 |
| IFI44 | 4.240521 | 5.042811 | 0.80229 | 0.000133 | 0.002614 |
| DCN | 7.10747 | 7.90418 | 0.79671 | 0.003678 | 0.020847 |
| CXADR | 5.660077 | 6.45669 | 0.796612 | 0.000107 | 0.002316 |
| TUSC3 | 6.044572 | 6.836439 | 0.791867 | 6.08E-05 | 0.00167 |
| TPM1 | 7.083107 | 7.874935 | 0.791828 | 7.23E-06 | 0.000467 |
| IER5 | 4.213598 | 5.003598 | 0.79 | 0.000225 | 0.00356 |
| MARCKS | 4.677398 | 5.467279 | 0.789881 | 2.32E-06 | 0.000256 |
| FAM129A | 3.632598 | 4.420899 | 0.788301 | 9.81E-08 | 4.81E-05 |
| FMO3 | 3.951471 | 4.739169 | 0.787699 | 0.000119 | 0.002464 |
| KLRB1 | 4.59331 | 5.378056 | 0.784745 | 0.000203 | 0.003322 |
| CD2 | 4.357631 | 5.140424 | 0.782792 | 8.55E-05 | 0.00205 |
| GLIPR1 | 3.642843 | 4.422077 | 0.779234 | 1.08E-05 | 0.00057 |
| TESC | 4.68385 | 5.456152 | 0.772303 | 0.000453 | 0.005542 |
| PLA2G4A | 3.304206 | 4.070509 | 0.766303 | 7.87E-07 | 0.00018 |
| HLA-B | 9.212414 | 9.977744 | 0.76533 | 0.000957 | 0.008834 |
| EMP3 | 5.407018 | 6.172266 | 0.765247 | 0.000957 | 0.008834 |
| FXYD5 | 4.66694 | 5.423135 | 0.756195 | 0.000203 | 0.003322 |
| AKAP12 | 6.904271 | 7.660279 | 0.756008 | 0.000728 | 0.007482 |
| PON2 | 6.612302 | 7.367093 | 0.754791 | 7.64E-05 | 0.001921 |
| LY75 | 4.076024 | 4.830386 | 0.754362 | 0.000453 | 0.005542 |
| LTB | 4.982192 | 5.734711 | 0.752519 | 0.001922 | 0.013872 |
| MYC | 5.887111 | 6.639465 | 0.752354 | 0.000133 | 0.002614 |
| CFD | 4.409211 | 5.156315 | 0.747104 | 0.000798 | 0.007939 |
| CXCL1 | 3.767498 | 4.513439 | 0.745941 | 0.002465 | 0.016294 |
| DCK | 3.968492 | 4.712077 | 0.743585 | 3.11E-06 | 0.000296 |
| PSMB8 | 5.746451 | 6.488857 | 0.742406 | 0.000119 | 0.002464 |
| TNMD | 4.078968 | 4.816545 | 0.737577 | 0.005004 | 0.025396 |
| ASNS | 4.038481 | 4.773211 | 0.73473 | 2.33E-05 | 0.00089 |
| CD44 | 4.495719 | 5.230346 | 0.734627 | 1.08E-06 | 0.000196 |
| VSIG4 | 4.945352 | 5.679921 | 0.734569 | 0.00125 | 0.010529 |
| SH2B3 | 5.651066 | 6.380995 | 0.729929 | 6.30E-06 | 0.000432 |
| FHL1 | 5.239711 | 5.967919 | 0.728207 | 4.82E-05 | 0.001441 |
| RRM2 | 3.893916 | 4.619838 | 0.725922 | 1.23E-05 | 0.000602 |
| ITGAM | 3.674907 | 4.400315 | 0.725408 | 1.72E-06 | 0.000222 |
| TUBB6 | 5.465072 | 6.189551 | 0.724479 | 0.003678 | 0.020847 |
| KDELC1 | 3.924052 | 4.646683 | 0.72263 | 4.14E-06 | 0.000345 |
| ISG20 | 4.068139 | 4.787536 | 0.719397 | 0.004638 | 0.024216 |
| RAC2 | 5.274462 | 5.990232 | 0.71577 | 0.000337 | 0.004604 |
| CCR2 | 3.556036 | 4.270197 | 0.714161 | 2.43E-07 | 9.54E-05 |
| CRISPLD2 | 5.972738 | 6.686459 | 0.713721 | 0.000874 | 0.008417 |
| HIST1H2BK | 5.523103 | 6.236653 | 0.71355 | 0.000411 | 0.005237 |
| MAFB | 5.619262 | 6.331254 | 0.711992 | 0.002465 | 0.016294 |
| VWF | 4.593736 | 5.299695 | 0.70596 | 1.08E-05 | 0.00057 |
| NCF2 | 3.855512 | 4.560459 | 0.704947 | 0.000373 | 0.004906 |
| CKLF | 5.252061 | 5.955508 | 0.703447 | 1.40E-05 | 0.000629 |
| BIRC3 | 3.623264 | 4.326362 | 0.703097 | 0.000603 | 0.006623 |
| PDGFRA | 5.869274 | 6.570595 | 0.701322 | 0.00125 | 0.010529 |
| RFTN1 | 5.778993 | 6.479964 | 0.700971 | 0.000249 | 0.003769 |
| NMI | 5.53389 | 6.233161 | 0.69927 | 1.40E-05 | 0.000629 |
| SACS | 3.147646 | 3.845804 | 0.698159 | 1.08E-06 | 0.000196 |
| VCAM1 | 8.526489 | 9.223939 | 0.69745 | 0.001145 | 0.009991 |
| NXN | 4.839585 | 5.535719 | 0.696134 | 6.69E-07 | 0.000173 |
| HLA-DMA | 6.628629 | 7.323931 | 0.695302 | 0.001766 | 0.013156 |
| GALNT7 | 5.435982 | 6.130287 | 0.694305 | 5.41E-05 | 0.001539 |
| GBP2 | 4.425039 | 5.118972 | 0.693934 | 0.001145 | 0.009991 |
| CXCR4 | 4.699139 | 5.39207 | 0.692931 | 0.001766 | 0.013156 |
| SCRN1 | 5.76816 | 6.460994 | 0.692834 | 6.30E-06 | 0.000432 |
| CHODL | 4.506461 | 5.196941 | 0.69048 | 4.82E-05 | 0.001441 |
| LPCAT1 | 5.159554 | 5.848787 | 0.689233 | 0.000499 | 0.005883 |
| LAMP3 | 3.57612 | 4.259272 | 0.683152 | 0.001622 | 0.012538 |
| DOCK2 | 3.529762 | 4.210491 | 0.680729 | 2.98E-05 | 0.001049 |
| TES | 5.127551 | 5.808012 | 0.680461 | 1.40E-05 | 0.000629 |
| LYN | 4.620718 | 5.300822 | 0.680105 | 9.46E-06 | 0.000534 |
| DKK3 | 3.630651 | 4.308859 | 0.678208 | 1.60E-05 | 0.000693 |
| ITM2C | 7.132987 | 7.809448 | 0.676461 | 0.000148 | 0.002767 |
| PTPRE | 4.072328 | 4.745353 | 0.673025 | 4.77E-06 | 0.000372 |
| FCER1G | 5.840763 | 6.513682 | 0.672919 | 0.004296 | 0.023069 |
| YWHAH | 5.898456 | 6.571161 | 0.672705 | 9.56E-05 | 0.00216 |
| FRZB | 4.41596 | 5.087748 | 0.671788 | 0.000603 | 0.006623 |
| MEST | 6.75029 | 7.42071 | 0.670421 | 0.000549 | 0.006219 |
| PFN1 | 6.71895 | 7.382369 | 0.663418 | 5.41E-05 | 0.001539 |
| TUBB | 7.222501 | 7.88536 | 0.662859 | 1.72E-06 | 0.000222 |
| PHLDA2 | 3.717189 | 4.379741 | 0.662552 | 0.002089 | 0.014694 |
| CD1C | 5.089594 | 5.750968 | 0.661374 | 7.23E-06 | 0.000467 |
| AEBP1 | 5.191906 | 5.845965 | 0.654059 | 0.000874 | 0.008417 |
| WNT5A | 3.39096 | 4.044695 | 0.653735 | 2.32E-06 | 0.000256 |
| SNAI2 | 4.365523 | 5.019196 | 0.653673 | 0.000453 | 0.005542 |
| PRC1 | 3.397616 | 4.051197 | 0.653581 | 3.37E-05 | 0.00114 |
| MNS1 | 4.434336 | 5.087381 | 0.653045 | 0.000663 | 0.006978 |
| AP1S2 | 5.110767 | 5.76244 | 0.651673 | 1.08E-06 | 0.000196 |
| PMP22 | 5.405747 | 6.054137 | 0.64839 | 0.000549 | 0.006219 |
| APOBEC3B | 3.366335 | 4.013672 | 0.647337 | 9.56E-05 | 0.00216 |
| BHLHE41 | 3.847773 | 4.49477 | 0.646997 | 7.23E-06 | 0.000467 |
| DDX60 | 5.078576 | 5.723261 | 0.644685 | 0.000373 | 0.004906 |
| NAP1L1 | 7.035919 | 7.678144 | 0.642225 | 2.69E-06 | 0.000278 |
| ISG15 | 5.638293 | 6.280242 | 0.641949 | 0.008356 | 0.036211 |
| TNFRSF11B | 6.075952 | 6.712984 | 0.637032 | 0.000119 | 0.002464 |
| KCTD12 | 6.532044 | 7.168546 | 0.636502 | 0.000798 | 0.007939 |
| RNASET2 | 6.456859 | 7.092263 | 0.635404 | 0.000107 | 0.002316 |
| DSE | 5.398771 | 6.032304 | 0.633533 | 0.000874 | 0.008417 |
| TM4SF1 | 5.11124 | 5.743397 | 0.632157 | 0.000164 | 0.002938 |
| ERAP2 | 3.700852 | 4.331987 | 0.631135 | 0.01031 | 0.041539 |
| SCG5 | 3.265603 | 3.896642 | 0.631039 | 0.000164 | 0.002938 |
| DNMT1 | 4.904147 | 5.533787 | 0.62964 | 2.64E-05 | 0.000952 |
| TUBA1B | 8.970318 | 9.599674 | 0.629356 | 5.41E-05 | 0.001539 |
| FBN1 | 3.873862 | 4.503176 | 0.629315 | 2.04E-07 | 8.33E-05 |
| ZEB2 | 3.188734 | 3.816615 | 0.627881 | 3.80E-05 | 0.001247 |
| ARPC5 | 7.308916 | 7.933613 | 0.624697 | 0.000337 | 0.004604 |
| ALOX5 | 3.658854 | 4.282781 | 0.623927 | 7.23E-06 | 0.000467 |
| IL7R | 4.186857 | 4.810704 | 0.623847 | 0.003976 | 0.021954 |
| IMPDH2 | 7.159839 | 7.783133 | 0.623294 | 9.46E-06 | 0.000534 |
| ADH1C | 3.988699 | 4.611884 | 0.623185 | 0.007781 | 0.034621 |
| CXCL12 | 6.922124 | 7.544634 | 0.62251 | 0.001365 | 0.011157 |
| FZD2 | 3.446431 | 4.068291 | 0.62186 | 2.64E-05 | 0.000952 |
| CLEC7A | 3.497961 | 4.119121 | 0.621159 | 0.000663 | 0.006978 |
| ECT2 | 3.079963 | 3.699562 | 0.619599 | 6.69E-07 | 0.000173 |
| CYP1B1 | 4.881271 | 5.500226 | 0.618955 | 0.000549 | 0.006219 |
| FCGR2B | 3.871068 | 4.489734 | 0.618666 | 0.002465 | 0.016294 |
| CRLF3 | 5.263502 | 5.882114 | 0.618612 | 6.82E-05 | 0.001803 |
| PSMB10 | 5.151303 | 5.767746 | 0.616443 | 0.004638 | 0.024216 |
| PPAP2C | 3.76417 | 4.380276 | 0.616106 | 2.00E-06 | 0.000239 |
| CST6 | 3.476038 | 4.091002 | 0.614964 | 0.000276 | 0.00403 |
| SPRY1 | 5.555704 | 6.168267 | 0.612563 | 2.00E-06 | 0.000239 |
| CLEC10A | 3.88541 | 4.4964 | 0.61099 | 0.00125 | 0.010529 |
| SOX4 | 6.212663 | 6.823313 | 0.61065 | 0.001365 | 0.011157 |
| CSGALNACT1 | 4.618078 | 5.227554 | 0.609476 | 0.000453 | 0.005542 |
| GPNMB | 5.942149 | 6.550902 | 0.608754 | 0.0034 | 0.01995 |
| B2M | 10.16676 | 10.77398 | 0.60722 | 7.64E-05 | 0.001921 |
| FABP4 | 3.735568 | 4.342379 | 0.606811 | 0.0034 | 0.01995 |
| IFNGR1 | 6.197216 | 6.803332 | 0.606116 | 0.000276 | 0.00403 |
| CPVL | 6.596097 | 7.201803 | 0.605706 | 0.001145 | 0.009991 |
| CPE | 5.005871 | 5.6106 | 0.604729 | 0.000225 | 0.00356 |
| CDH11 | 4.790631 | 5.394947 | 0.604315 | 0.00125 | 0.010529 |
| KRT18 | 8.353321 | 8.956771 | 0.60345 | 0.000798 | 0.007939 |
| COL4A2 | 6.254248 | 6.857223 | 0.602975 | 0.002899 | 0.017966 |
| TMEM123 | 6.748497 | 7.349169 | 0.600672 | 0.000411 | 0.005237 |
| SFN | 5.726741 | 6.325269 | 0.598529 | 0.004296 | 0.023069 |
| MMD | 5.647432 | 6.245056 | 0.597624 | 0.001047 | 0.009425 |
| HLA-DMB | 6.661212 | 7.257343 | 0.596131 | 0.007781 | 0.034621 |
| P2RY14 | 3.208828 | 3.804876 | 0.596049 | 0.000119 | 0.002464 |
| SERPINE2 | 7.856741 | 8.451268 | 0.594527 | 0.000728 | 0.007482 |
| DACT1 | 3.856768 | 4.45118 | 0.594412 | 8.55E-05 | 0.00205 |
| PRKCB | 3.723475 | 4.315169 | 0.591694 | 2.33E-05 | 0.00089 |
| IGSF6 | 3.069477 | 3.658697 | 0.58922 | 0.000164 | 0.002938 |
| NLGN4X | 3.008408 | 3.595019 | 0.586611 | 1.42E-07 | 6.64E-05 |
| EZH2 | 3.039007 | 3.623611 | 0.584603 | 1.26E-06 | 0.0002 |
| CTSK | 4.851363 | 5.435866 | 0.584502 | 0.000874 | 0.008417 |
| TDO2 | 3.481438 | 4.064278 | 0.58284 | 7.81E-05 | 0.001959 |
| FAS | 5.085662 | 5.667668 | 0.582006 | 4.14E-06 | 0.000345 |
| TLR7 | 3.027097 | 3.606222 | 0.579125 | 6.30E-06 | 0.000432 |
| CCND2 | 3.953903 | 4.532463 | 0.57856 | 1.60E-08 | 3.15E-05 |
| PNMAL1 | 4.975352 | 5.552248 | 0.576896 | 0.000305 | 0.004322 |
| GPR18 | 3.227313 | 3.803298 | 0.575985 | 0.000276 | 0.00403 |
| EFNB2 | 4.71445 | 5.289672 | 0.575222 | 7.23E-06 | 0.000467 |
| HMGB2 | 6.04959 | 6.624522 | 0.574933 | 0.011822 | 0.045683 |
| ROBO1 | 4.140031 | 4.713932 | 0.573902 | 0.000276 | 0.00403 |
| KPNA2 | 6.106224 | 6.677901 | 0.571678 | 0.000549 | 0.006219 |
| LCP2 | 3.405855 | 3.977087 | 0.571232 | 6.82E-05 | 0.001803 |
| PECAM1 | 5.389219 | 5.960119 | 0.5709 | 0.002674 | 0.017135 |
| RTP4 | 4.419378 | 4.989897 | 0.570518 | 0.000957 | 0.008834 |
| RNASE1 | 7.532015 | 8.102211 | 0.570196 | 0.001766 | 0.013156 |
| FZD7 | 4.112488 | 4.682451 | 0.569963 | 2.33E-05 | 0.00089 |
| RPS3 | 9.713973 | 10.28378 | 0.569804 | 6.82E-05 | 0.001803 |
| CORO1A | 4.3683 | 4.937571 | 0.569271 | 0.007241 | 0.032889 |
| PFKP | 6.064399 | 6.63309 | 0.568691 | 0.000957 | 0.008834 |
| GZMB | 3.374426 | 3.943055 | 0.568629 | 0.012648 | 0.047931 |
| ACSL4 | 3.243553 | 3.811636 | 0.568083 | 0.000276 | 0.00403 |
| FZD6 | 5.542116 | 6.109803 | 0.567686 | 0.000148 | 0.002767 |
| CDC7 | 3.418237 | 3.984908 | 0.566671 | 4.28E-05 | 0.001355 |
| ARHGEF6 | 5.04034 | 5.604485 | 0.564145 | 9.56E-05 | 0.00216 |
| SLC26A2 | 4.042094 | 4.605135 | 0.563041 | 3.37E-05 | 0.00114 |
| BLNK | 5.927611 | 6.488132 | 0.560521 | 0.003976 | 0.021954 |
| DOK5 | 5.268278 | 5.827399 | 0.559121 | 0.000107 | 0.002316 |
| MEIS2 | 4.874509 | 5.433013 | 0.558504 | 0.000728 | 0.007482 |
| ESYT1 | 6.192213 | 6.749683 | 0.55747 | 2.33E-05 | 0.00089 |
| FLRT3 | 6.871202 | 7.428308 | 0.557106 | 0.008968 | 0.037859 |
| ID1 | 6.794683 | 7.351135 | 0.556452 | 0.006258 | 0.029505 |
| ARL6IP5 | 7.674646 | 8.231056 | 0.55641 | 2.06E-05 | 0.000817 |
| GALNT1 | 5.857935 | 6.414059 | 0.556124 | 9.46E-06 | 0.000534 |
| CAPG | 6.417474 | 6.9708 | 0.553326 | 0.000453 | 0.005542 |
| HN1 | 5.71116 | 6.264288 | 0.553128 | 0.000249 | 0.003769 |
| GPR65 | 3.313775 | 3.866669 | 0.552893 | 0.000183 | 0.003155 |
| GPR126 | 4.978296 | 5.529445 | 0.55115 | 4.82E-05 | 0.001441 |
| GIMAP6 | 4.138122 | 4.687035 | 0.548913 | 0.01031 | 0.041539 |
| EFHC1 | 4.087262 | 4.635666 | 0.548404 | 9.46E-06 | 0.000534 |
| TNFAIP6 | 3.297585 | 3.845848 | 0.548263 | 9.46E-06 | 0.000534 |
| CLEC4A | 3.341965 | 3.888564 | 0.546599 | 1.23E-05 | 0.000602 |
| PTN | 4.707986 | 5.254304 | 0.546318 | 0.006258 | 0.029505 |
| CD14 | 5.900725 | 6.446525 | 0.5458 | 0.01031 | 0.041539 |
| SPON1 | 4.535509 | 5.081127 | 0.545619 | 0.000499 | 0.005883 |
| FCHSD2 | 4.79832 | 5.343881 | 0.545561 | 0.000133 | 0.002614 |
| LIPG | 4.08939 | 4.633285 | 0.543895 | 0.005004 | 0.025396 |
| XBP1 | 8.054048 | 8.597798 | 0.543751 | 0.000183 | 0.003155 |
| DEK | 7.151265 | 7.695005 | 0.543739 | 7.64E-05 | 0.001921 |
| CD97 | 4.509487 | 5.053089 | 0.543602 | 0.001766 | 0.013156 |
| PLCB4 | 3.480661 | 4.024186 | 0.543525 | 8.55E-05 | 0.00205 |
| ADCY7 | 3.268994 | 3.811029 | 0.542035 | 9.46E-06 | 0.000534 |
| TMPRSS4 | 5.29031 | 5.831486 | 0.541176 | 0.000148 | 0.002767 |
| MMP2 | 5.138371 | 5.678561 | 0.54019 | 0.000107 | 0.002316 |
| FCN1 | 3.890023 | 4.429591 | 0.539568 | 0.003976 | 0.021954 |
| GIMAP4 | 6.023309 | 6.561811 | 0.538502 | 0.003141 | 0.01888 |
| GABRP | 3.29732 | 3.833224 | 0.535904 | 1.81E-05 | 0.000757 |
| PTPLA | 3.986411 | 4.520191 | 0.53378 | 9.56E-05 | 0.00216 |
| MID1 | 4.488239 | 5.020298 | 0.532059 | 0.000148 | 0.002767 |
| CD38 | 4.506606 | 5.038385 | 0.531778 | 0.001622 | 0.012538 |
| CAV2 | 4.792742 | 5.322419 | 0.529677 | 0.000453 | 0.005542 |
| PRSS23 | 6.526799 | 7.055701 | 0.528902 | 0.001047 | 0.009425 |
| PERP | 5.075434 | 5.604181 | 0.528747 | 0.000874 | 0.008417 |
| ITM2A | 5.202573 | 5.730158 | 0.527585 | 0.000798 | 0.007939 |
| AHR | 5.223227 | 5.750159 | 0.526932 | 2.32E-06 | 0.000256 |
| PRKX | 3.747694 | 4.274121 | 0.526427 | 0.000249 | 0.003769 |
| CCNB1 | 3.753221 | 4.279156 | 0.525935 | 0.000203 | 0.003322 |
| ANXA5 | 7.82302 | 8.348558 | 0.525538 | 0.000411 | 0.005237 |
| TMEM158 | 3.940159 | 4.465691 | 0.525532 | 0.000337 | 0.004604 |
| KCNJ8 | 4.600188 | 5.124772 | 0.524584 | 0.001922 | 0.013872 |
| IQGAP1 | 6.140547 | 6.6635 | 0.522953 | 1.81E-05 | 0.000757 |
| RAB27A | 5.095429 | 5.615521 | 0.520092 | 0.000305 | 0.004322 |
| KIAA0101 | 4.199601 | 4.718647 | 0.519046 | 0.000305 | 0.004322 |
| LIPA | 7.395437 | 7.914126 | 0.518689 | 0.005004 | 0.025396 |
| MX2 | 4.505016 | 5.023303 | 0.518287 | 0.001047 | 0.009425 |
| REV3L | 4.476946 | 4.993841 | 0.516895 | 0.000133 | 0.002614 |
| KDELR3 | 4.518026 | 5.034812 | 0.516787 | 9.23E-07 | 0.000196 |
| RHOA | 8.629626 | 9.146086 | 0.51646 | 4.28E-05 | 0.001355 |
| HLA-F | 7.871078 | 8.387483 | 0.516405 | 0.000728 | 0.007482 |
| LCK | 3.792443 | 4.308134 | 0.515691 | 1.81E-05 | 0.000757 |
| IL33 | 3.044506 | 3.559501 | 0.514995 | 1.23E-05 | 0.000602 |
| C2CD2 | 5.146282 | 5.659453 | 0.513171 | 2.06E-05 | 0.000817 |
| S100A10 | 8.810149 | 9.322579 | 0.51243 | 0.001622 | 0.012538 |
| S100A11 | 7.364554 | 7.876753 | 0.512199 | 0.009618 | 0.0397 |
| CD9 | 7.778645 | 8.290021 | 0.511376 | 0.006258 | 0.029505 |
| SFRP1 | 6.131701 | 6.64212 | 0.510419 | 0.003678 | 0.020847 |
| NFE2L3 | 3.568239 | 4.078619 | 0.51038 | 2.06E-05 | 0.000817 |
| TRAF5 | 4.107304 | 4.615563 | 0.508259 | 6.82E-05 | 0.001803 |
| LPGAT1 | 4.031773 | 4.53937 | 0.507596 | 0.000957 | 0.008834 |
| PLAT | 5.106747 | 5.614301 | 0.507554 | 0.003976 | 0.021954 |
| COMP | 3.387135 | 3.894524 | 0.507389 | 0.001145 | 0.009991 |
| ACSL5 | 4.352116 | 4.859281 | 0.507165 | 1.47E-06 | 0.000219 |
| INTS8 | 5.873601 | 6.378726 | 0.505125 | 0.000411 | 0.005237 |
| MELK | 3.798811 | 4.303779 | 0.504968 | 7.23E-06 | 0.000467 |
| MX1 | 5.71354 | 6.215047 | 0.501507 | 0.011044 | 0.043549 |
| COLEC12 | 3.812637 | 4.313959 | 0.501322 | 0.000119 | 0.002464 |
| ADAM10 | 4.8132 | 5.314369 | 0.501168 | 2.64E-05 | 0.000952 |
| HMGB3 | 5.114524 | 5.615199 | 0.500675 | 3.37E-05 | 0.00114 |
| ACTR3 | 7.6513 | 8.151655 | 0.500354 | 0.000874 | 0.008417 |
| SPATA2L | 6.17841 | 5.678278 | -0.50013 | 0.000133 | 0.002614 |
| AUH | 8.098726 | 7.597143 | -0.50158 | 0.002899 | 0.017966 |
| LHPP | 6.460668 | 5.958325 | -0.50234 | 0.000549 | 0.006219 |
| PGPEP1 | 6.744461 | 6.240983 | -0.50348 | 0.000133 | 0.002614 |
| NR1I3 | 4.544803 | 4.039835 | -0.50497 | 0.012648 | 0.047931 |
| ZNF91 | 7.268451 | 6.763265 | -0.50519 | 0.011822 | 0.045683 |
| LIME1 | 6.751402 | 6.244883 | -0.50652 | 0.00227 | 0.015466 |
| ALAD | 6.4729 | 5.963529 | -0.50937 | 0.003976 | 0.021954 |
| KLHL3 | 6.382034 | 5.872591 | -0.50944 | 0.000874 | 0.008417 |
| PNPLA4 | 6.773646 | 6.262328 | -0.51132 | 2.64E-05 | 0.000952 |
| ARG2 | 7.071544 | 6.559698 | -0.51185 | 0.000499 | 0.005883 |
| EPHX1 | 7.380032 | 6.866317 | -0.51372 | 0.01031 | 0.041539 |
| EFHD1 | 8.849824 | 8.334822 | -0.515 | 0.000164 | 0.002938 |
| SLC12A6 | 5.557176 | 5.04132 | -0.51586 | 0.001145 | 0.009991 |
| HAGH | 7.72242 | 7.205432 | -0.51699 | 0.000957 | 0.008834 |
| ECHDC2 | 8.77297 | 8.255712 | -0.51726 | 0.001766 | 0.013156 |
| RENBP | 6.351364 | 5.833305 | -0.51806 | 0.000411 | 0.005237 |
| DUSP1 | 6.090937 | 5.565902 | -0.52504 | 0.006258 | 0.029505 |
| NECAB2 | 6.400768 | 5.875117 | -0.52565 | 0.000276 | 0.00403 |
| RGS7 | 4.085062 | 3.557666 | -0.5274 | 0.000203 | 0.003322 |
| TTC38 | 7.999196 | 7.471397 | -0.5278 | 0.000119 | 0.002464 |
| CYP46A1 | 5.191868 | 4.662274 | -0.52959 | 0.001047 | 0.009425 |
| SIGIRR | 7.787891 | 7.255273 | -0.53262 | 0.008968 | 0.037859 |
| KLF9 | 5.609855 | 5.076071 | -0.53378 | 3.37E-05 | 0.00114 |
| PCSK1N | 7.01931 | 6.483548 | -0.53576 | 0.007781 | 0.034621 |
| GADD45G | 4.391378 | 3.855137 | -0.53624 | 0.000337 | 0.004604 |
| TCEAL2 | 5.182725 | 4.645103 | -0.53762 | 0.003678 | 0.020847 |
| DLST | 6.815669 | 6.275065 | -0.5406 | 2.06E-05 | 0.000817 |
| SLC39A4 | 7.178759 | 6.637903 | -0.54086 | 0.000798 | 0.007939 |
| NR4A2 | 3.850147 | 3.307567 | -0.54258 | 0.001766 | 0.013156 |
| RAB11FIP3 | 7.072585 | 6.528111 | -0.54447 | 0.000107 | 0.002316 |
| CYP2C8 | 3.727279 | 3.179666 | -0.54761 | 0.000549 | 0.006219 |
| SLIT2 | 6.898819 | 6.350555 | -0.54826 | 0.000874 | 0.008417 |
| CLIC5 | 5.443724 | 4.890002 | -0.55372 | 0.002674 | 0.017135 |
| PROC | 6.703572 | 6.148556 | -0.55502 | 0.011044 | 0.043549 |
| HDHD3 | 6.748908 | 6.193101 | -0.55581 | 0.001766 | 0.013156 |
| FXYD2 | 9.379134 | 8.820649 | -0.55849 | 0.00125 | 0.010529 |
| CLDN8 | 7.476388 | 6.914873 | -0.56152 | 0.000203 | 0.003322 |
| MAPT | 6.25387 | 5.691281 | -0.56259 | 7.64E-05 | 0.001921 |
| SLC2A4RG | 6.484422 | 5.916225 | -0.5682 | 5.41E-05 | 0.001539 |
| CSDC2 | 5.966293 | 5.397349 | -0.56894 | 0.000107 | 0.002316 |
| PTGER3 | 5.796581 | 5.223352 | -0.57323 | 8.12E-08 | 4.42E-05 |
| SALL1 | 5.616191 | 5.041383 | -0.57481 | 0.000337 | 0.004604 |
| ALDH1L1 | 8.107834 | 7.532578 | -0.57526 | 0.0034 | 0.01995 |
| ACADL | 6.111162 | 5.535267 | -0.57589 | 0.00227 | 0.015466 |
| ISOC2 | 7.507557 | 6.931229 | -0.57633 | 0.000164 | 0.002938 |
| FMO5 | 6.10494 | 5.52817 | -0.57677 | 0.005812 | 0.028105 |
| UGT2B28 | 8.807427 | 8.230323 | -0.5771 | 0.007241 | 0.032889 |
| ACP5 | 8.097183 | 7.515361 | -0.58182 | 0.003678 | 0.020847 |
| CYFIP2 | 7.652966 | 7.069859 | -0.58311 | 0.000249 | 0.003769 |
| APOLD1 | 4.560492 | 3.976383 | -0.58411 | 0.000798 | 0.007939 |
| SPAG5 | 6.57756 | 5.992966 | -0.58459 | 0.002674 | 0.017135 |
| PDK2 | 6.615742 | 6.02957 | -0.58617 | 4.77E-06 | 0.000372 |
| SFXN1 | 6.875683 | 6.283309 | -0.59237 | 9.56E-05 | 0.00216 |
| STRA6 | 6.190022 | 5.595385 | -0.59464 | 0.000373 | 0.004906 |
| SLC12A3 | 6.721801 | 6.126413 | -0.59539 | 0.002465 | 0.016294 |
| PTH1R | 8.702335 | 8.103512 | -0.59882 | 0.00227 | 0.015466 |
| SERPINF2 | 6.288564 | 5.688563 | -0.6 | 0.004638 | 0.024216 |
| QDPR | 9.416322 | 8.815514 | -0.60081 | 0.004638 | 0.024216 |
| DUSP9 | 5.509019 | 4.90532 | -0.6037 | 0.000305 | 0.004322 |
| FGF9 | 6.246324 | 5.636171 | -0.61015 | 0.000663 | 0.006978 |
| SLC5A2 | 7.790134 | 7.177852 | -0.61228 | 0.003678 | 0.020847 |
| CD83 | 6.497734 | 5.885276 | -0.61246 | 0.001365 | 0.011157 |
| ACAA1 | 8.48558 | 7.87232 | -0.61326 | 0.001047 | 0.009425 |
| METTL1 | 6.563594 | 5.947648 | -0.61595 | 1.23E-05 | 0.000602 |
| CRYM | 9.128766 | 8.510499 | -0.61827 | 0.001766 | 0.013156 |
| FABP3 | 7.405758 | 6.786566 | -0.61919 | 0.003976 | 0.021954 |
| KHK | 8.267128 | 7.645633 | -0.6215 | 0.002899 | 0.017966 |
| HSD17B14 | 6.806568 | 6.18367 | -0.6229 | 0.000203 | 0.003322 |
| SLC46A3 | 7.029856 | 6.401526 | -0.62833 | 5.41E-05 | 0.001539 |
| GLB1L2 | 7.410689 | 6.780603 | -0.63009 | 0.000337 | 0.004604 |
| FMO4 | 8.039851 | 7.408788 | -0.63106 | 0.01031 | 0.041539 |
| PBLD | 9.250809 | 8.619172 | -0.63164 | 0.012648 | 0.047931 |
| HPN | 7.728739 | 7.096569 | -0.63217 | 0.001488 | 0.011832 |
| FOLH1 | 6.428403 | 5.792998 | -0.63541 | 0.005395 | 0.026664 |
| ZFP36 | 6.890013 | 6.254355 | -0.63566 | 0.00227 | 0.015466 |
| GCH1 | 7.647405 | 7.010857 | -0.63655 | 0.002899 | 0.017966 |
| ETNK2 | 6.005611 | 5.367292 | -0.63832 | 0.000874 | 0.008417 |
| RNF186 | 7.491132 | 6.852287 | -0.63885 | 0.005812 | 0.028105 |
| ARSF | 6.59988 | 5.958731 | -0.64115 | 0.000549 | 0.006219 |
| PC | 7.179833 | 6.536717 | -0.64312 | 0.000874 | 0.008417 |
| ALDH8A1 | 8.714093 | 8.069979 | -0.64411 | 0.00227 | 0.015466 |
| MT1G | 11.24677 | 10.60159 | -0.64518 | 0.000798 | 0.007939 |
| AKR7A3 | 8.750015 | 8.100876 | -0.64914 | 0.01031 | 0.041539 |
| AGXT | 6.153257 | 5.504069 | -0.64919 | 0.007241 | 0.032889 |
| PPP1R1A | 6.9127 | 6.262146 | -0.65055 | 0.008356 | 0.036211 |
| ACOT7 | 7.870142 | 7.217787 | -0.65236 | 0.001047 | 0.009425 |
| ACSM5 | 6.456904 | 5.804379 | -0.65252 | 0.000305 | 0.004322 |
| TSKU | 6.828629 | 6.173523 | -0.65511 | 5.49E-06 | 0.000399 |
| ENTPD5 | 7.066697 | 6.395248 | -0.67145 | 0.000549 | 0.006219 |
| MAOA | 8.228523 | 7.556813 | -0.67171 | 0.004296 | 0.023069 |
| ACADSB | 6.931638 | 6.252272 | -0.67937 | 0.004638 | 0.024216 |
| PPP1R16B | 7.010976 | 6.328833 | -0.68214 | 0.000305 | 0.004322 |
| C11orf71 | 6.189566 | 5.506608 | -0.68296 | 1.60E-05 | 0.000693 |
| ACSF2 | 8.042246 | 7.358607 | -0.68364 | 0.001922 | 0.013872 |
| PLCL1 | 6.625098 | 5.938351 | -0.68675 | 0.008968 | 0.037859 |
| MT1X | 10.14113 | 9.452384 | -0.68875 | 0.000663 | 0.006978 |
| PXMP2 | 8.058033 | 7.366739 | -0.69129 | 6.08E-05 | 0.00167 |
| HRSP12 | 9.904808 | 9.212074 | -0.69273 | 0.011044 | 0.043549 |
| PIPOX | 8.733471 | 8.035614 | -0.69786 | 0.008356 | 0.036211 |
| CA2 | 9.368695 | 8.666371 | -0.70232 | 0.011822 | 0.045683 |
| PCK1 | 10.07727 | 9.368664 | -0.70861 | 0.003678 | 0.020847 |
| CIDEB | 7.32805 | 6.617261 | -0.71079 | 7.64E-05 | 0.001921 |
| HMGCS2 | 6.879259 | 6.167263 | -0.712 | 0.009618 | 0.0397 |
| EAF2 | 7.466642 | 6.753288 | -0.71335 | 0.000225 | 0.00356 |
| HSD11B2 | 8.07005 | 7.354203 | -0.71585 | 4.82E-05 | 0.001441 |
| MT1F | 10.11979 | 9.403691 | -0.7161 | 0.000549 | 0.006219 |
| SGK2 | 6.624073 | 5.905551 | -0.71852 | 4.82E-05 | 0.001441 |
| MT1M | 6.324939 | 5.602288 | -0.72265 | 0.01031 | 0.041539 |
| GPC5 | 5.153139 | 4.427284 | -0.72586 | 0.008968 | 0.037859 |
| ALDH4A1 | 8.037927 | 7.308518 | -0.72941 | 0.000957 | 0.008834 |
| CYP4F3 | 7.420757 | 6.69006 | -0.7307 | 0.012648 | 0.047931 |
| USP2 | 5.629743 | 4.894727 | -0.73502 | 4.28E-05 | 0.001355 |
| XPNPEP2 | 7.926711 | 7.185076 | -0.74163 | 0.011044 | 0.043549 |
| MACROD1 | 6.421471 | 5.676788 | -0.74468 | 4.82E-05 | 0.001441 |
| HYAL1 | 8.009837 | 7.261509 | -0.74833 | 0.000119 | 0.002464 |
| ASB9 | 6.119016 | 5.370209 | -0.74881 | 6.30E-06 | 0.000432 |
| NELL1 | 5.32483 | 4.573741 | -0.75109 | 2.69E-06 | 0.000278 |
| FBP1 | 9.333041 | 8.580693 | -0.75235 | 0.001766 | 0.013156 |
| CPNE6 | 5.000522 | 4.245428 | -0.75509 | 0.000728 | 0.007482 |
| ECHDC3 | 8.731136 | 7.975064 | -0.75607 | 0.000133 | 0.002614 |
| HPGD | 6.735117 | 5.977738 | -0.75738 | 0.007241 | 0.032889 |
| CYP4A11 | 6.579671 | 5.816729 | -0.76294 | 0.000411 | 0.005237 |
| DNMT3L | 5.600592 | 4.831874 | -0.76872 | 0.000499 | 0.005883 |
| SLC7A7 | 9.93097 | 9.155331 | -0.77564 | 0.001622 | 0.012538 |
| SERPINA6 | 7.306094 | 6.517717 | -0.78838 | 0.005812 | 0.028105 |
| ALDOB | 11.391 | 10.5964 | -0.7946 | 0.001365 | 0.011157 |
| GADD45A | 8.685398 | 7.881105 | -0.80429 | 9.56E-05 | 0.00216 |
| CALML3 | 5.321156 | 4.50779 | -0.81337 | 7.64E-05 | 0.001921 |
| GSTA1 | 10.03066 | 9.215196 | -0.81546 | 0.005004 | 0.025396 |
| MME | 8.664505 | 7.840571 | -0.82393 | 0.008968 | 0.037859 |
| DAO | 7.890689 | 7.064128 | -0.82656 | 0.006734 | 0.031178 |
| SH3GL2 | 6.393685 | 5.565446 | -0.82824 | 0.002899 | 0.017966 |
| LPL | 5.061537 | 4.232686 | -0.82885 | 0.000337 | 0.004604 |
| ANK2 | 7.733315 | 6.903722 | -0.82959 | 0.008968 | 0.037859 |
| FOLH1B | 5.924425 | 5.093318 | -0.83111 | 0.003678 | 0.020847 |
| ADM | 7.628064 | 6.79492 | -0.83314 | 0.000203 | 0.003322 |
| GLYAT | 8.858801 | 8.02388 | -0.83492 | 0.000453 | 0.005542 |
| CEL | 4.542728 | 3.690854 | -0.85187 | 8.12E-08 | 4.42E-05 |
| GHR | 6.4071 | 5.523102 | -0.884 | 0.000225 | 0.00356 |
| FTCD | 7.546708 | 6.655204 | -0.8915 | 0.001488 | 0.011832 |
| APOC3 | 5.567468 | 4.661649 | -0.90582 | 0.000305 | 0.004322 |
| PRODH2 | 9.257065 | 8.350857 | -0.90621 | 0.004296 | 0.023069 |
| RHCG | 6.901309 | 5.993905 | -0.9074 | 0.000133 | 0.002614 |
| DEFB1 | 10.1393 | 9.231153 | -0.90815 | 4.14E-06 | 0.000345 |
| KCNN2 | 4.843963 | 3.933281 | -0.91068 | 5.41E-05 | 0.001539 |
| GADD45B | 6.688494 | 5.758754 | -0.92974 | 4.14E-06 | 0.000345 |
| KL | 8.754252 | 7.824038 | -0.93021 | 0.008968 | 0.037859 |
| G0S2 | 7.525416 | 6.582632 | -0.94278 | 3.59E-06 | 0.000323 |
| HRG | 7.051828 | 6.083284 | -0.96854 | 1.47E-06 | 0.000219 |
| THY1 | 8.317791 | 7.345646 | -0.97214 | 0.000337 | 0.004604 |
| PYROXD1 | 8.171515 | 7.191013 | -0.9805 | 0.002899 | 0.017966 |
| TYRP1 | 5.714983 | 4.728513 | -0.98647 | 0.000249 | 0.003769 |
| SLC34A1 | 7.544592 | 6.544448 | -1.00014 | 0.000663 | 0.006978 |
| UMOD | 10.64172 | 9.641033 | -1.00069 | 0.012085 | 0.046679 |
| RALYL | 6.463498 | 5.449826 | -1.01367 | 8.28E-06 | 0.000504 |
| NPHS2 | 6.513598 | 5.492971 | -1.02063 | 0.000957 | 0.008834 |
| ASPA | 7.925501 | 6.891126 | -1.03437 | 0.000225 | 0.00356 |
| KNG1 | 9.111583 | 8.076847 | -1.03474 | 0.000183 | 0.003155 |
| APOH | 6.966639 | 5.926327 | -1.04031 | 0.011822 | 0.045683 |
| PVALB | 6.423026 | 5.353166 | -1.06986 | 6.82E-05 | 0.001803 |
| ATF3 | 5.644837 | 4.55379 | -1.09105 | 0.012648 | 0.047931 |
| UPB1 | 7.150508 | 6.03125 | -1.11926 | 0.000305 | 0.004322 |
| AFM | 6.909811 | 5.760434 | -1.14938 | 0.006258 | 0.029505 |
| KLK1 | 6.941099 | 5.713046 | -1.22805 | 1.26E-06 | 0.0002 |
| GC | 5.480851 | 4.227033 | -1.25382 | 0.0034 | 0.01995 |
| CYP27B1 | 6.709323 | 5.453923 | -1.2554 | 0.001488 | 0.011832 |
| FOSB | 5.393591 | 3.999568 | -1.39402 | 0.000164 | 0.002938 |
| EGF | 7.231429 | 5.63792 | -1.59351 | 4.77E-06 | 0.000372 |
| G6PC | 6.745907 | 5.109812 | -1.63609 | 2.00E-06 | 0.000239 |
