# Supplementary material for: Integrated Analysis of Multiple Microarray Studies to Identify Core Gene-Expression Signatures Involved in Tubulointerstitial Injury in Diabetic Nephropathy
Source: Biomed Res Int. 2022 May 10;2022:9554658. doi: 10.1155/2022/9554658 (PMC9113875; doi:10.1155/2022/9554658)
Supplement: Supplementary Materials — Figure S1: the boxplot figure before or after removing batch in GSE99325 and GSE104954. Table S1: 545 DEGS. Table S2: the number of genes and the identified hub genes in each coexpression module. Table S3 related genes the top 30 nodes with neighbors and expanded ranked by degree method in “cyto-hubba” of PPI network. Table S4: KEGG analysis of green module genes. [file 9554658.f1.zip › Table S2.docx]

Table S2 The number of genes and the identified hub genes in each co-expression module

| Module | Gene count | Hub genes |
| --- | --- | --- |
| Green | 347 | VCAN; ANXA1; ANXA2; C1S; TGFBI; LYZ; COL3A1; COL1A2; FSTL1; LAPTM5; PYCARD; HOPX; TIMP1; RAB31; IRF8; ARHGDIB; DNMT1; PLAC8; ITGAM; ITGB2; BIRC3; EVI2A; EVI2B; RGS10; IFI16; PSMB9; HCLS1; TNFAIP8; PTPRE; PTPRC; CASP1; SRGN; IL10RA; TPBG; CD53; CD48; CD44; CSTA; CTSS; MS4A6A; |
| Blue | 134 | PC; XPNPEP2; ECHDC3; PXMP2; FBP1; AFM; DAO; FTCD; UGT2B28; KHK; MME; HAGH; PIPOX; HPGD; CIDEB; ACAA1; ACSF2; GLYAT; PRODH2; SLC34A1; PLP2; QDPR; ALDH1L1; HDHD3; ALAD; ASB9; ASPA; HSD17B14; GSTA1; CRYM; UPB1; |
| Grey | 64 | - |
